# ERMP1 Exerts Tumor‐Suppressive Functions in KIRC by Inhibiting PI3K/AKT Signaling and Remodeling the Immune Microenvironment: A Pan‐Cancer Analysis

**DOI:** 10.1155/humu/7717815

**Published:** 2026-04-07

**Authors:** Ziyang Liu, Jiahao Shan, Tao Yang, Qiang Zhang, Lianghong Ma, Feilong Yang

**Affiliations:** ^1^ First Clinical Medical College, Ningxia Medical University, Yinchuan, Ningxia, China, nxmu.edu.cn; ^2^ Department of Urology, General Hospital of Ningxia Medical University, Yinchuan, Ningxia, China, nxmu.edu.cn

**Keywords:** ERMP1, kidney renal clear cell carcinoma (kirc), PI3K/AKT signaling pathway, tumor microenvironment, tumor suppressor

## Abstract

**Background:**

Kidney renal clear cell carcinoma (KIRC) is an aggressive malignancy with limited therapeutic options, highlighting the need for novel biomarkers and therapeutic targets. Although endoplasmic reticulum metallopeptidase 1 (ERMP1) has been implicated in cancer progression, its specific role, clinical significance, and underlying mechanisms in KIRC remain poorly defined.

**Methods:**

We integrated data from the TCGA, GTEx, and GEO databases to conduct a pan‐cancer analysis, aiming to systematically evaluate the expression patterns, genetic alterations, and prognostic value of ERMP1. Single‐cell transcriptomic data were utilized to decipher its cell‐type–specific expression within the tumor immune microenvironment. By establishing ERMP1 overexpression models in KIRC cell lines (Caki‐1 and A498), we assessed its impact on malignant phenotypes using CCK‐8, colony formation, transwell, and wound healing assays. The underlying mechanisms were further investigated via Western blotting. Finally, by establishing a xenograft tumor model in vivo, we evaluated the inhibitory effect of ERMP1 on tumor growth of KIRC in vivo.

**Results:**

The prognostic value of ERMP1 exhibits cancer type‐specificity. In KIRC, its high expression serves as an independent marker for favorable prognosis and is negatively correlated with advanced pathological features. Single‐cell analysis revealed that ERMP1 is enriched in regulatory T cells and proliferative exhausted T cells. Its high expression is closely associated with an immunologically activated tumor microenvironment, characterized by upregulation of immunostimulatory factors and chemokines, alongside increased lymphocyte infiltration. Functionally, ERMP1 overexpression significantly suppressed the proliferation, migration, invasion, and clonogenic ability of KIRC cells. Mechanistically, ERMP1 inhibits the PI3K/AKT signaling pathway, impedes epithelial‐mesenchymal transition (manifested as E‐cadherin upregulation and N‐cadherin downregulation), and reduces the expression of invasion‐related proteins (MMP2 and MMP9) and cell cycle‐related proteins (Cyclin D1 and CDK4). In vivo xenograft tumor assays confirmed that ERMP1 overexpression could significantly inhibit tumor growth.

**Conclusion:**

This study confirms that ERMP1 exhibits significant potential in tumorigenesis, diagnosis, prognosis, and regulation of the tumor microenvironment (TME). In KIRC, ERMP1 may exert tumor‐suppressive effects by inhibiting the PI3K/AKT signaling pathway and regulating immune responses, thus representing a potential prognostic biomarker and therapeutic target.

## 1. Introduction

KIRC, the most prevalent subtype of renal cell carcinoma, constitutes approximately 80% of cases and is notably invasive and metastatic [1–3]. Although surgical and targeted therapies have advanced [4, 5], early detection of KIRC remains difficult [4]. As a result, the majority of patients are diagnosed at advanced stages and has a poor prognosis, with a 5‐year survival rate below 15% [6–8]. Thus, identifying key molecules involved in KIRC pathogenesis and elucidating their mechanisms of action are critical for improving patient outcomes.

Endoplasmic reticulum (ER) stress and the ensuing unfolded protein response (UPR) are commonly activated in cancer [9, 10]. This activation promotes tumorigenesis and progression and is strongly linked to therapy resistance, highlighting its potential as a therapeutic target [11]. Endoplasmic reticulum metallopeptidase 1 (ERMP1), an ER‐resident zinc‐binding protease of the M28 peptidase family, participates in the UPR and cellular stress responses [12, 13]. As a key regulator of ER stress, ERMP1 localizes to the 9p24 amplification region, which has been associated with the pathogenesis of several cancers [14]. Previous studies indicate that ERMP1 is upregulated in various tumors, including breast and colorectal cancer, and may facilitate tumor survival by modulating ER stress [14–17]. In clear cell renal cell carcinoma (ccRCC), ERMP1 has been identified as part of an 18‐RNA network associated with survival [18]. Nevertheless, its expression pattern, prognostic significance, and precise functional mechanisms in KIRC remain incompletely understood, and whether it exerts cancer type‐specific roles remains to be fully elucidated.

The ER represents a critical interface between tumor and immune activity; UPR activation can coordinate both prosurvival and immunosuppressive pathways, thereby fostering an inhibitory TME [19, 20]. Emerging evidence suggests that ERMP1 may contribute to immune regulation [12, 21]. As an ER stress‐related differentially expressed gene, ERMP1 constitutes one component of a prognostic risk score model in osteosarcoma. Its expression is elevated in metastatic osteosarcoma and correlates with immune cell infiltration—including plasma cells and CD8^+^ T cells—in the TME, indirectly influencing immune‐related prognosis in osteosarcoma [4, 22]. However, a systematic investigation of ERMP1′s expression profile and its correlation with immune cell subsets in the KIRC TME is still lacking.

Given this context, this study integrates multiomics data with in vitro experiments to clarify the expression profile, prognostic relevance, and potential mechanisms of ERMP1 in KIRC. We first examined ERMP1 expression in KIRC tissues using public databases such as TCGA and GTEx and evaluated its relationship with clinicopathological parameters and patient survival. Further analyses were performed to assess the correlation between ERMP1 expression and immune cell infiltration as well as immune modulator expression, exploring its potential influence on the TME. Finally, through in vitro overexpression experiments, we investigated the role of ERMP1 in regulating malignant phenotypes of renal cancer cells and preliminarily examined whether its function involves the PI3K/AKT signaling pathway.

Our work not only establishes ERMP1 as a potential independent prognostic biomarker in KIRC, but also unveils its possible functional mechanisms through immunomodulation of the TME and involvement in key signaling pathways, thereby providing fresh experimental evidence for understanding the specific role of ERMP1 in KIRC.

## 2. Materials and Methods

### 2.1. Data Sources and Preprocessing

The standardized pan‐cancer dataset (including TCGA, TARGET, and GTEx; project accession number: phs000178.v12.p8) was downloaded from UCSC Xena (v2023‐09). Differential expression analysis was performed using the limma package (v3.58.0) in R (v4.3.1), with the screening thresholds set at |log_2_(fold change)| > 1 and adjusted *p* value (adj. p) < 0.05. Somatic mutation, copy number variation (CNV), and gene fusion data were sourced from cBioPortal (https://www.cbioportal.org), whereas GISTIC‐processed gene‐level CNV data were acquired from the GDC portal (https://portal.gdc.cancer.gov/). Expression values were log_2_(x + 0.001) transformed and batch effects were corrected using ComBat‐seq.

### 2.2. Pan‐Cancer Analysis of ERMP1

ERMP1 expression between tumor and normal tissues was compared using the Wilcoxon rank‐sum test. Associations between ERMP1 expression and immunotypes, molecular subtypes, clinical stage, and tumor grade across cancers were evaluated via the TISIDB database. Patients were stratified by median ERMP1 expression into high‐ and low‐expression groups. Kaplan–Meier survival analysis with the log‐rank test was applied to overall survival (OS), disease‐specific survival (DSS), and progression‐free interval (PFI) data. Spearman′s correlation assessed the relationship between ERMP1 expression and tumor stage/grade in KIRC, USC, and STAD. Diagnostic performance was evaluated using ROC curve analysis. A multivariate Cox model incorporating ERMP1 expression and clinical variables was used to construct a nomogram predicting 3‐ and 5‐year survival, with calibration curves assessing accuracy. GO and KEGG enrichment analyses were performed on ERMP1‐coexpressed genes.

### 2.3. Expression of ERMP1 in the Tumor Immune Microenvironment (TIME)

Using the KIRC single‐cell RNA‐seq dataset GSE121636 (GEO), ERMP1 expression was analyzed across immune cell subsets (e.g., T cells, dendritic cells, macrophages, and NK cells) and stromal cells. Expression heterogeneity among immune subsets was evaluated by one‐way ANOVA, and an unpaired *t*‐test compared expression between immune and stromal cells. Differences in immune subset proportions between ERMP1‐positive and ERMP1‐negative samples were examined. A significance threshold of *p* < 0.001 was used in this section.

### 2.4. Association of ERMP1 With the Immune Microenvironment in KIRC

Transcriptomic data from the TCGA‐KIRC cohort were used to investigate ERMP1′s role in the immune microenvironment. Spearman′s correlation analyzed associations between ERMP1 and immunostimulators (e.g., CD40 and CD80), chemokines, and HLA molecules. GSEA validated enrichment of ERMP1‐associated genes in immune‐related signatures, including BCR/TCR diversity and IFN‐*γ* response. Immune cell infiltration levels were estimated using CIBERSORT and correlated with ERMP1 expression. The tumor‐infiltrating lymphocyte (MeTIL) score was compared between ERMP1 high‐ and low‐expression groups (split by median) using the Wilcoxon rank‐sum test.

### 2.5. Ethics Statement and Tissue Specimens

This study was approved by the Research Ethics Committee of the General Hospital of Ningxia Medical University (Approval No. K021/2021). A total of 60 ccRCC tissues and 60 matched nontumorous kidney specimens were collected from patients who underwent surgery at the General Hospital of Ningxia Medical University. All patients had complete clinical records and follow‐up data and had not received any form of tumor‐specific therapy before diagnosis. Tumors were staged according to the 8th edition of the AJCC TNM staging system and histologically classified per the World Health Organization (WHO) criteria for renal tumors. Written informed consent was obtained from each participant.

### 2.6. RNA Extraction and qRT‐PCR

Total RNA was extracted from tissues or cultured cells using the FastPure RNA Isolation Kit (Vazyme) according to the manufacturer′s instructions. RNA concentration and purity were determined spectrophotometrically. First‐strand cDNA was synthesized from 1 *μ*g of total RNA using the Evo M‐MLV RT Premix Kit (Accurate Biotechnology). qRT‐PCR was subsequently performed using the SYBR Green Premix Pro Taq HS qPCR Kit (Accurate Biotechnology) on a real‐time PCR detection system. The primer sequences used were as follows:

ERMP1 forward: 5 ^′^‐ CACAGAGGACCTGGTGGATG ‐3 ^′^

ERMP1 reverse: 5 ^′^‐ TGGTAGCCACAGTGACGGAT ‐3 ^′^

GAPDH forward: 5 ^′^‐ TGACTTCAACAGCGACACCC ‐3 ^′^

GAPDH reverse: 5 ^′^‐ CACCCTGTTGCTGTAGCCAA ‐3 ^′^.

ERMP1 expression was normalized to GAPDH using the 2^*ΔΔ*Ct^ method.

### 2.7. Western Blot Analysis

Tissues and cells were lysed in RIPA buffer (Beyotime) containing protease and phosphatase inhibitors. Total protein concentrations were determined by the bicinchoninic acid (BCA) assay. Equal amounts of protein (30–50 *μ*g) were resolved on 6%–12% SDS–PAGE gels and transferred to PVDF membranes. Membranes were blocked with 5% nonfat milk in PBST (PBS+0.1% Tween‐20) for 1 h at room temperature, then incubated overnight at 4°C with primary antibodies. After washing, membranes were incubated with HRP‐conjugated secondary antibodies for 1 h at room temperature. Blots were developed with ECL substrate and imaged. Densitometric analysis was performed using ImageJ v1.8.0 (NIH). All experiments were performed in triplicate. Primary antibodies and dilutions: ERMP1 (rabbit; Proteintech, 27224‐1‐AP; WB 1:2000; and IHC 1:100); Phospho‐Akt (Ser473) (rabbit; CST, 4060 T; and WB 1:2000); Akt (rabbit; Proteintech, 10176‐2‐AP; and WB 1:2000); Phospho‐PI3K (rabbit; Abmart, T40065S; and WB 1:2000); PI3K (rabbit; Abmart, T40064S; and WB 1:2000); N‐Cadherin (rabbit; Affinity, AF4039; and WB 1:2000); E‐Cadherin (rabbit; Affinity, AF0131; and WB 1:2000); MMP2 (rabbit; Proteintech, 10373‐2‐AP; and WB 1:2000); MMP9 (rabbit; Proteintech, 10375‐2‐AP; and WB 1:2000); Cyclin D1 (mouse; Proteintech, 60186‐1‐Ig; and WB 1:2000); CDK4 (rabbit; Proteintech, 11026‐1‐AP; and WB 1:2000); GAPDH (rabbit; Affinity, AF7021; and WB 1:2000).

### 2.8. Immunohistochemistry (IHC)

IHC staining was performed according to standard procedures. Tissue sections were deparaffinized in xylene, rehydrated through a graded ethanol series, and subjected to antigen retrieval using citrate buffer via microwave heating. Endogenous peroxidase activity was blocked with 3% hydrogen peroxide, followed by incubation with 10% normal goat serum for 30 min at room temperature for blocking. The sections were then incubated with primary antibodies overnight at 4°C. After washing with PBS the next day, the sections were incubated with corresponding secondary antibodies for 2 h at room temperature, followed by color development with DAB and nuclear counterstaining with hematoxylin. ERMP1 staining was semiquantitatively scored by two independent observers. The total IHC score (ranging from 0 to 6) was calculated by summing the staining intensity score (0 = *none*; 1 = *weak*; 2 = *moderate*; and 3 = *strong*) and the percentage of positive cells score (0% = 0%; 1% ≤ 10%; 2% = 10%–50%; 3%≥50%). A total score < 4 was defined as low ERMP1 expression, whereas a score ≥ 4 was defined as high expression. Adjacent nontumor renal tissues were used as controls.

### 2.9. Cell Culture

The human renal cell carcinoma cell lines Caki‐1 (derived from a penile metastasis of a male patient) and A498 (derived from a renal adenocarcinoma of a female patient) were obtained from the Cell Bank of the Chinese Academy of Sciences (Shanghai, China) in 2023. Both cell lines were authenticated using short tandem repeat (STR) profiling within 1 year prior to these experiments, which showed a match with the reference profiles (Caki‐1: RRID:CVCL_0234; A498: RRID:CVCL_1056). The cell lines were confirmed to be free of mycoplasma contamination using a PCR‐based detection method and have not been previously reported as misidentified or contaminated. Cells were maintained in high‐glucose DMEM medium supplemented with 10% fetal bovine serum (FBS) and 1% penicillin‐streptomycin (10 *μ*g/mL) at 37°C in a humidified atmosphere containing 5% CO_2_. Cells were passaged at 70%–80% confluence, and the culture medium was replaced every 2–3 days.

#### 2.9.1. Lentivirus‐Mediated ERMP1 Overexpression

To investigate the gain‐of‐function effects of ERMP1 in KIRC, ERMP1‐overexpressing lentivirus (LV‐ERMP1) and empty vector control lentivirus (LV‐Vector) constructed by Shanghai GeneChem Co. Ltd. were used. Caki‐1 and A498 cells were seeded into 6‐well plates. When cell confluence reached 60%–70%, the viral supernatant was added at the recommended multiplicity of infection (MOI) along with 5 *μ*g/mL Polybrene. The medium was replaced with complete culture medium 8 h after infection. At 48 h postinfection, the medium was changed to complete medium containing an appropriate concentration of puromycin (Solarbio) for selection, which continued for 7–10 days until all cells in the control group had died. The surviving polyclonal cell pool was collected, expanded, and used for subsequent experiments.

### 2.10. Establishment of Xenograft Tumor Models in Mice

Male BALB/c nude mice aged 5–6 weeks were purchased from Changzhou Cavens Laboratory Animal Co. Ltd. (Guangzhou, China). Mice were housed in an SPF (specific pathogen free) animal facility under the following conditions: temperature 22°C–26°C, relative humidity 50%–60%, 12 h light/12 h dark cycle, with free access to food and water. After 1 week of acclimatization, mice were randomly assigned using a random number table into two groups of four mice each: the Vector control group and the ERMP1 overexpression group. One day prior to the experiment, logarithmic‐phase vector empty vector control A498 cells and A498 cells stably overexpressing ERMP1 were digested with trypsin, centrifuged, resuspended in serum‐free RPMI‐1640 medium, and adjusted to a concentration of 1 × 10^7^ cells/mL. Using a sterile syringe, 100 *μ*L of cell suspension (containing 1 × 10^6^ cells) was slowly injected subcutaneously beneath the right costal margin of each mouse to establish the xenograft model. At the experimental endpoint (4 weeks postinjection), mice were euthanized by cervical dislocation. Subcutaneous tumor tissue was completely excised and photographed for documentation. Tumor weight was then precisely measured using an electronic balance for subsequent statistical analysis.

### 2.11. CCK‐8 Assay

Cell viability was assessed using the Cell Counting Kit‐8 (CCK‐8; Sigma‐Aldrich, 96992). Cells were seeded in 96‐well plates at a density of 6000 cells per well and cultured overnight. Then, 10 *μ*L of CCK‐8 reagent was added to each well, followed by incubation for 2 h at 37°C. The absorbance at 450 nm was measured using a synergy microplate reader (BioTek Instruments, Inc.). The experiment was conducted with three technical replicates per group and independently repeated three times.

### 2.12. Wound Healing and Colony Formation Assays

For the wound healing assay, cells were seeded in 6‐well plates (1 × 10^6^ cells/well) and cultured to full confluence. After pretreatment with 10 *μ*g/mL mitomycin C (S8146, Selleck) for 2 h to inhibit proliferation, a scratch was created using a 200 *μ*L pipette tip. The cells were then washed with PBS and cultured in serum‐free medium. Images were captured at 0 and 24 h under an inverted microscope (4× objective). The wound closure rate was calculated as: (Initial wound area—remaining wound area at 24 h)/initial wound area × 100%.

For the colony formation assay, 1–2 × 10^3^ cells were seeded per well in 6‐well plates (*n* = 3 replicates per group) and cultured for 2 weeks. The resulting colonies were fixed with 4% paraformaldehyde for 20 min, stained with 0.5% crystal violet for 10 min, washed, photographed, and counted.

### 2.13. Transwell Migration and Invasion Assay

Cell migration was assessed using transwell chambers. Cells (1 × 10^5^) suspended in serum‐free medium were seeded into the upper chamber, whereas the lower chamber was filled with medium containing 20% FBS as a chemoattractant. After incubation at 37°C for 24 h, the cells were fixed with 4% paraformaldehyde and stained with 0.1% crystal violet. Following air‐drying, the migrated cells were observed under a microscope. For each sample, at least three random fields were photographed, and the number of migrated cells was counted for statistical analysis.

### 2.14. Statistical Analysis

Statistical analyses were primarily performed using R (v3.6.3), with GraphPad Prism 8.0 and SPSS 27.0 employed for visualization. Data are presented as mean ± standard deviation. Group comparisons for continuous variables utilized the Wilcoxon rank‐sum test or Student′s *t*‐test. Categorical variables were analyzed by chi‐square test, whereas correlations between continuous variables were assessed using Spearman′s method. Survival analysis was conducted with Kaplan–Meier curves and log‐rank tests. Time‐dependent ROC analysis was performed using the R package “timeROC”. Univariate and multivariate Cox proportional hazards models were applied to identify independent prognostic factors; variables with *p* < 0.05 in univariate analysis were included in the multivariate analysis. Nomogram calibration was evaluated visually. The significance threshold was set at *p* < 0.05, unless otherwise specified (e.g., *p* < 0.001 in TIME analyses).

## 3. Results

### 3.1. Genetic Alterations and Expression Pattern of ERMP1 Across Cancer Types

To systematically investigate the somatic alteration landscape of the ERMP1 gene across cancers, we conducted an integrated genomic analysis of 33 TCGA cancer types. Results revealed widespread somatic alterations in ERMP1, including point mutations, amplifications, deep deletions, and multiple alterations. CNVs, particularly gene deletions, represented the predominant alteration type. To clarify the distribution patterns of ERMP1 alterations across different cancer types, we compared their frequencies. ERMP1 exhibited the highest alteration frequency in stomach adenocarcinoma (STAD), characterized by co‐occurrence of mutations, amplifications, and deletions (Figure 1a). Furthermore, it showed relatively high alteration rates in colorectal adenocarcinoma (COAD) and uterine corpus endometrial carcinoma (UCEC), whereas no significant mutations were detected in cholangiocarcinoma (CHOL). To further decipher the potential functional impact of ERMP1 mutations, we mapped and annotated the protein mutation sites. Protein structural analysis identified M28 as a recurrent mutation hotspot. Notably, we identified 93 missense mutations, 18 truncating mutations, and seven splice variants densely clustered near the transmembrane domain and signal peptide region (Figure 1b), strongly suggesting this region as a functional hotspot for ERMP1.

Figure 1Genetic alterations and expression pattern of ERMP1 across cancer types. (a) Alteration frequency in TCGA cancers. Analysis of ERMP1 genetic alterations across 33 cancer types (red: mutations; blue: amplifications; purple: deep deletions; gray: multiple alterations). (b) Protein structure and mutation distribution. Domain architecture includes signal peptide (yellow) and transmembrane domains (green). The M28 residue (arrow) represents a recurrent mutation hotspot.  ^∗∗^p < 0.01 and  ^∗∗∗^ *p* < 0.001.

**Figure:**
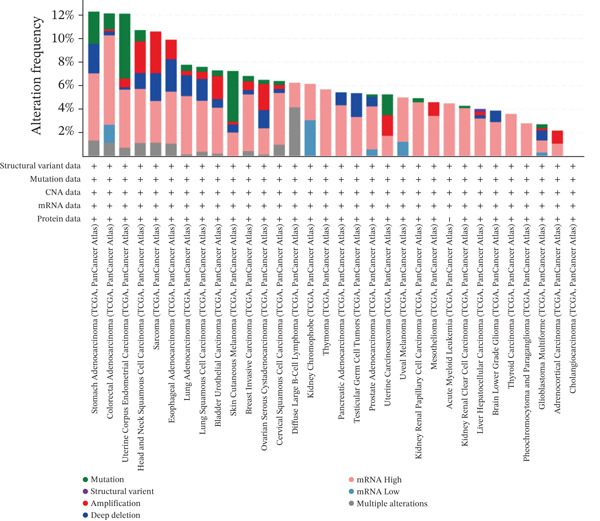
(a)

**Figure:**
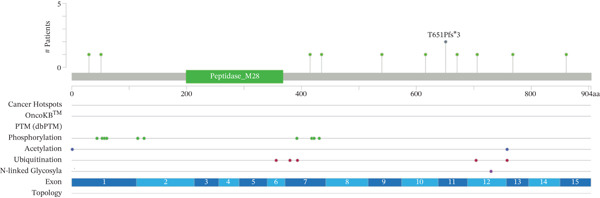
(b)

### 3.2. Integrated Multidatabase Analysis Reveals Pan‐Cancer Expression Heterogeneity of ERMP1

To investigate the expression patterns of ERMP1 in different cancers, we first performed a comparative analysis between cancerous and normal tissues using the TIMER database. The results revealed that compared with corresponding normal tissues, ERMP1 was significantly upregulated in 11 cancer types (e.g., BRCA, CESC, CHOL; fold change > 1.5, FDR < 0.05) and significantly downregulated in six other malignancies (e.g., GBM, HNSC; fold change < 0.67, FDR < 0.01) (Figures 2a and S1). To further validate and extend these findings, we integrated TCGA and GTEx datasets to utilize a broader range of normal tissues as controls for verification analysis. This comprehensive analysis confirmed widespread dysregulation of ERMP1 expression, demonstrating significant upregulation in 20 cancer types (including COAD, ESCA, and STAD) and significant downregulation in eight other cancers (e.g., ACC, KIRC, and LAML) (Figure 2b).

Figure 2Differential expression of ERMP1 between tumor and normal tissues. (a) TIMER database analysis shows significantly altered ERMP1 expression in various cancer types compared with adjacent normal tissues. (b) Integrated TCGA‐GTEx analysis reveals ERMP1 mRNA expression differences across 33 cancer types. ^∗^ *p* < 0.05;  ^∗∗^ *p* < 0.01;  ^∗∗∗^ *p* < 0.001.

**Figure:**
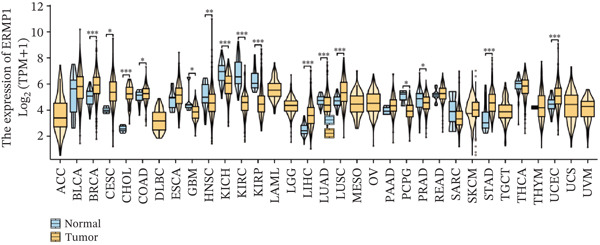
(a)

**Figure:**
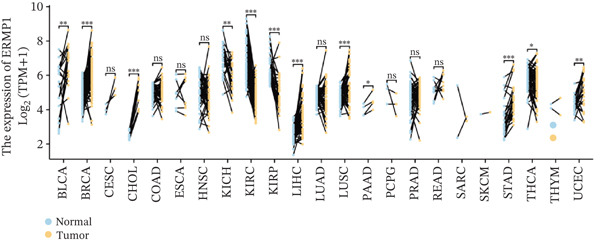
(b)

### 3.3. Expression Characteristics of ERMP1 in Immune Cell Subsets

To investigate the cellular localization of ERMP1 within the TIME, we first analyzed the distribution of its mRNA across different immune cell subsets. The results revealed significant heterogeneity in ERMP1 expression among various immune cells (Figure 3a,b). Notably, regulatory T cells (Tregs) and proliferative exhausted T cells (Tprolif) exhibited significantly higher ERMP1 expression levels compared with other subsets, indicating distinct cell type‐specific expression patterns. To validate this specific high‐expression pattern of ERMP1 in Treg and Tprolif cells, we performed analysis using an independent dataset (KIRC‐GSE121636). This validation confirmed that the average ERMP1 expression in Tprolif and Treg subsets remained consistently elevated compared with other immune populations (Figure 3c,d), strongly supporting preferential ERMP1 expression in these two T‐cell subtypes. To determine ERMP1 expression preference among major TIME components, we compared its levels in immune cells versus stromal cells. Analysis demonstrated significantly higher ERMP1 expression in immune cells compared with stromal cells (Figure 3e), suggesting its functional relevance is primarily associated with immune regulation. To further explore its functional impact, we correlated ERMP1 expression with immune cell composition. We found that the proportion of Tprolif cells was significantly higher in ERMP1‐high samples compared with ERMP1‐low samples (Figure 3f), linking its molecular expression to the remodeling of immune subset composition within the tumor microenvironment (TME).

Figure 3ERMP1 expression exhibits specificity within immune cells of the tumor microenvironment. (a, b) Scatter plots showing heterogeneous ERMP1 mRNA expression across immune cell subsets. *p* Values were determined by one‐way ANOVA. (c, d) Bar graphs validating the preferential expression of ERMP1 in Tprolif and Treg subsets using the KIRC‐GSE121636 dataset. Data are presented as mean ± SEM. (e) ERMP1 expression is significantly elevated in immune cells compared with stromal cells (unpaired *t*‐test). (f) Stacked bar plot revealing an increased proportion of Tprolif cells in ERMP1‐positive samples relative to ERMP1‐negative samples. Treg, regulatory T cell; Tprolif, proliferative exhausted T cell.

**Figure:**
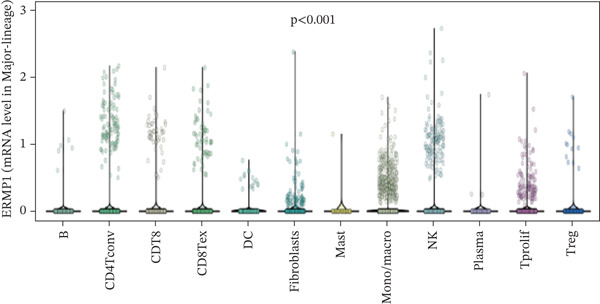
(a)

**Figure:**
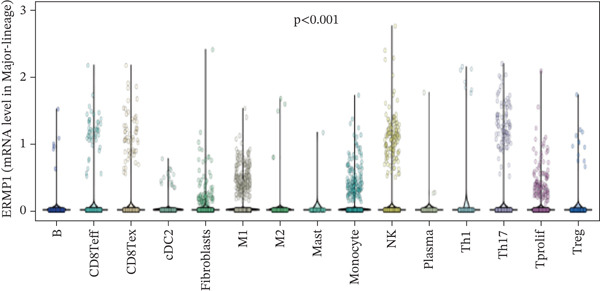
(b)

**Figure:**
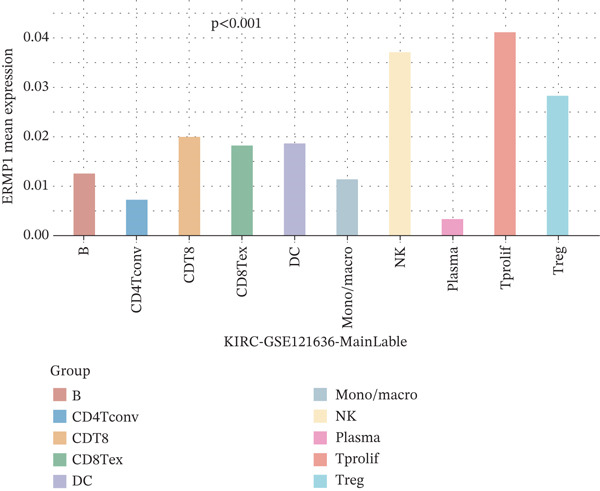
(c)

**Figure:**
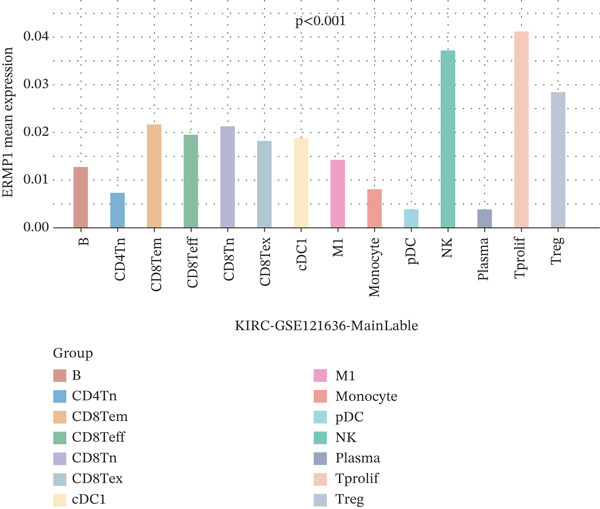
(d)

**Figure:**
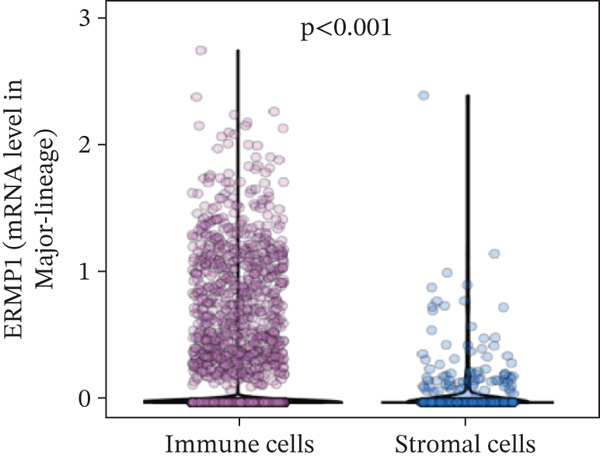
(e)

**Figure:**
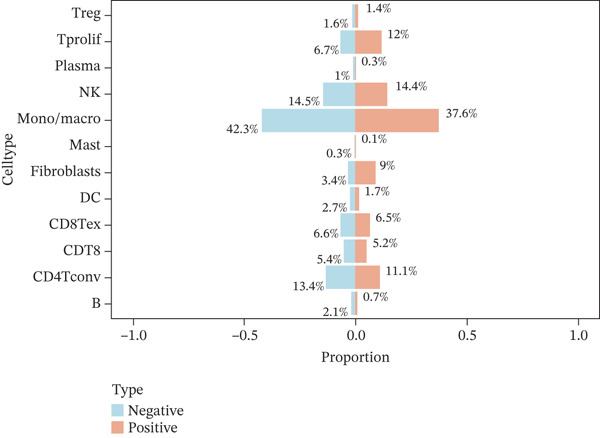
(f)

### 3.4. Prognostic Value of ERMP1 in Pan‐Cancer and Its Correlation With Tumor Stage/Grade

To evaluate the prognostic significance of ERMP1 expression, we performed survival analysis across multiple malignancies. Results demonstrated that the prognostic value of ERMP1 expression exhibits remarkable cancer type‐specificity (Figures 4a, 4b, and 4c). In KIRC, high ERMP1 expression was significantly associated with longer DSS (HR = 0.396, *p* = 1.31 × 10^−5^) and progression‐free survival (PFS; HR = 0.477, *p* = 1 × 10^−5^), suggesting a protective role. Conversely, in cancers such as hepatocellular carcinoma (LIHC) and breast cancer (BRCA), high ERMP1 expression correlated with increased patient mortality risk, revealing its heterogeneous prognostic effects across different cancers. To investigate the relationship between ERMP1 expression and tumor progression, we analyzed its association with tumor stage. In KIRC, ERMP1 expression levels significantly decreased with advancing tumor stage (Figure 4d,e). Further analysis of clinicopathological characteristics confirmed that low ERMP1 expression was significantly associated with more advanced disease features, including higher pathological T stage, M stage, and overall pathological stage (Table 1). This negative correlation was consistently observed in advanced uterine carcinosarcoma (USC; rho = −0.315, *p* = 0.031; Figure 4f) and high‐grade STAD (rho = −0.157, *p* = 0.015; Figure 4g). To determine ERMP1′s potential in distinguishing tumor from normal tissues, we evaluated its diagnostic performance across different cancers. Results showed that ERMP1 exhibited exceptionally high discriminatory accuracy for cervical cancer (CESC) and CHOL, with area under the receiver operating characteristic curve (AUC) values reaching 0.93 and 0.91, respectively. Furthermore, ERMP1 demonstrated moderate diagnostic value (AUC > 0.7) in six other cancer types including breast cancer (BRCA; Figure S2).

Figure 4Prognostic value of ERMP1 expression across cancers and its correlation with tumor stage/grade. (a–c) Forest plots showing the association between ERMP1 expression and patient survival in various cancer types. (a) Disease‐specific survival (DSS). (b) Overall survival (OS). (c) Progress‐free interval (PFI). Hazard ratios (HR) with 95% confidence intervals and *p* values are shown for each analysis. (d–g) Box plots illustrating the correlation of ERMP1 mRNA expression with tumor stage or grade. ERMP1 expression is significantly inversely correlated with advanced tumor stage in (d, e) kidney renal clear cell carcinoma (KIRC) (Spearman *ρ* = −0.236, *p* = 3.87 × 10^−8^ and *ρ* = −0.264, *p* = 0.0172, respectively), (f) uterine carcinosarcoma (USC) (*ρ* = −0.315, *p* = 0.031), and with high tumor grade in (g) stomach adenocarcinoma (STAD) (*ρ* = −0.157, *p* = 0.015).

**Figure:**
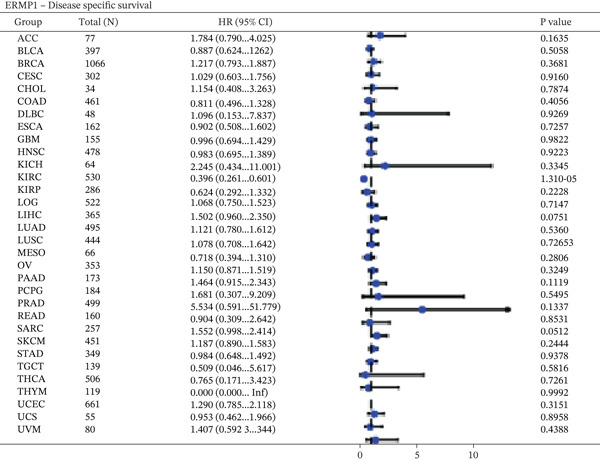
(a)

**Figure:**
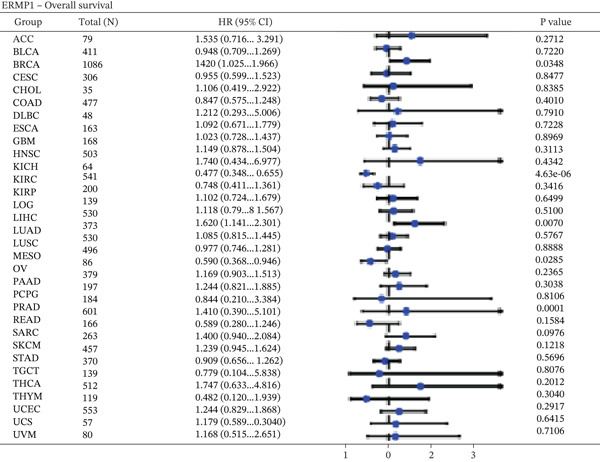
(b)

**Figure:**
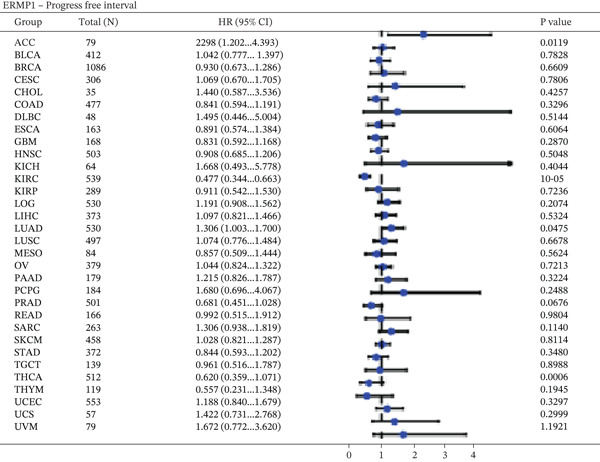
(c)

**Figure:**
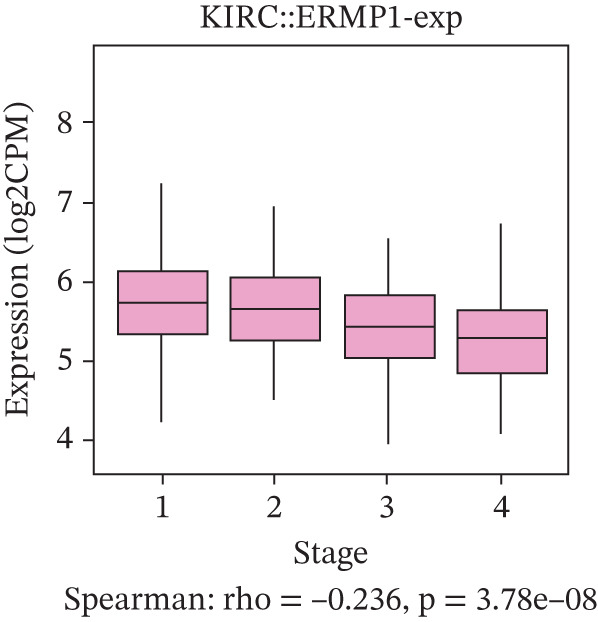
(d)

**Figure:**
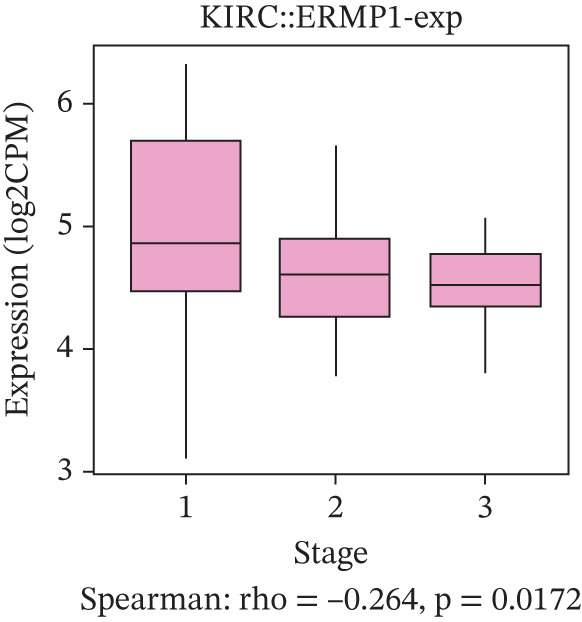
(e)

**Figure:**
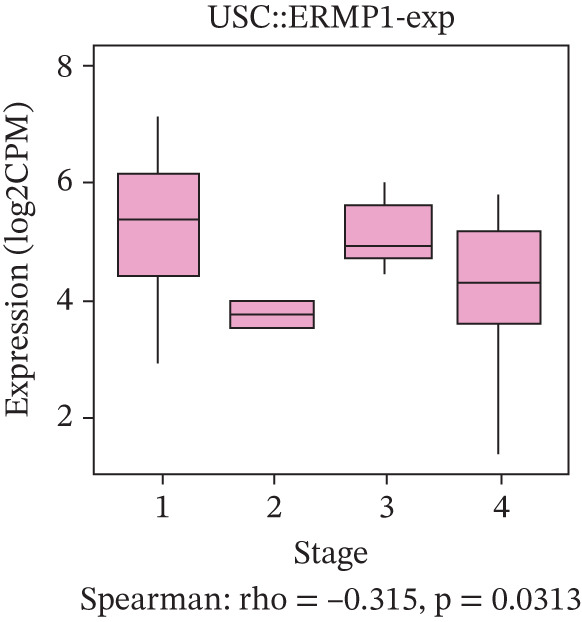
(f)

**Figure:**
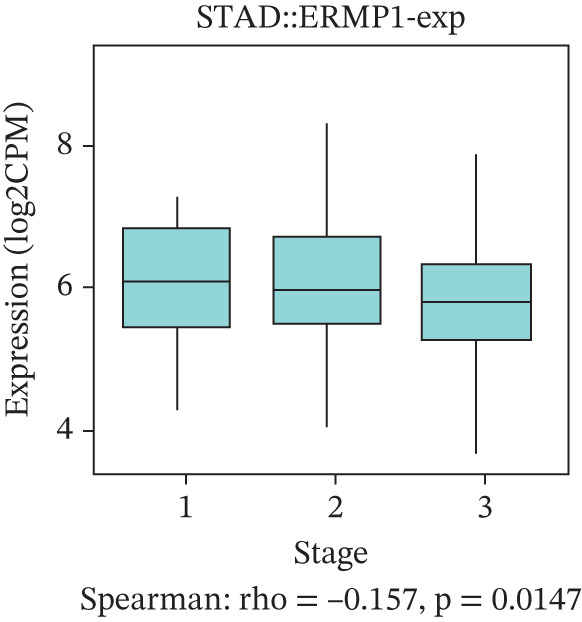
(g)

**Table 1:** Correlation between ERMP1 protein expression and clinicopathological characteristics in ccRCC patients.

Characteristics	Low expression of ERMP1	High expression of ERMP1	*p*
n	270	271	
Age, *n* (%)			0.112
≤ 60	125 (23.1%)	144 (26.6%)	
> 60	145 (26.8%)	127 (23.5%)	
Gender, *n* (%)			**0.026**
Female	81 (15%)	106 (19.6%)	
Male	189 (34.9%)	165 (30.5%)	
Pathologic T stage, *n* (%)			**< 0.001**
T1 and T2	151 (27.9%)	199 (36.8%)	
T3 and T4	119 (22%)	72 (13.3%)	
Pathologic *N* stage, *n* (%)			0.606
N0	120 (46.5%)	122 (47.3%)	
N1	9 (3.5%)	7 (2.7%)	
Pathologic M stage, n (%)			**< 0.001**
M0	202 (39.8%)	227 (44.7%)	
M1	54 (10.6%)	25 (4.9%)	
Pathologic stage, *n* (%)			**< 0.001**
Stage I and Stage II	136 (25.3%)	196 (36.4%)	
Stage III and Stage IV	132 (24.5%)	74 (13.8%)	

*Note:* The bolded values denote statistically significant results (e.g., values meeting the predefined significance threshold of *p* < 0.05, *p* < 0.01, or other established statistical criteria).

### 3.5. ERMP1 Shapes the TIME by Regulating Immune Molecule Expression, Immune Response Pathways, and Immune Cell Infiltration

To investigate the potential regulatory role of ERMP1 in the immune microenvironment of KIRC, we first analyzed its expression correlation with key immune molecules. Results showed that high ERMP1 expression was significantly associated with upregulation of various immune stimulators (e.g., CD40 and CD80), chemokines, and HLA molecules (e.g., HLA‐A and HLA‐B) (Figure 5a), suggesting ERMP1 may participate in enhancing intratumoral immune activation and antigen presentation processes. To further clarify the relationship between ERMP1 and the overall antitumor immune status, we evaluated its correlation with multiple immune response signatures. ERMP1 expression showed significant positive correlations with several features characterizing an immunologically active microenvironment, including BCR/TCR diversity, IFN‐*γ* response, and lymphocyte infiltration score (Figure 5b), indicating its close association with a more robust antitumor immune response. To dissect ERMP1′s impact at the cellular level, we used computational algorithms to assess its relationship with immune cell infiltration levels. In KIRC, ERMP1 expression positively correlated with infiltration levels of naive B cells and macrophages, but its association with various T cell subsets displayed diversity (Figure 5c). Furthermore, the tumor‐infiltrating lymphocyte (MeTIL) score was significantly higher in the ERMP1‐high group compared with the ERMP1‐low group (Figure 5d), confirming its consistency with enhanced overall lymphocyte infiltration. Comprehensive heat map analysis further validated extensive associations between ERMP1 and various immune cell subsets as well as cytotoxic activity (Figure 5e).

Figure 5ERMP1 expression is associated with an immunologically active tumor microenvironment in KIRC.(a) Heatmap showing the upregulation of immunostimulatory factors, chemokines, and HLA molecules in tumors with high ERMP1 expression.(b) Heatmap of immune and genomic features. High ERMP1 expression correlates with an active immune phenotype, including increased BCR/TCR diversity, IFN‐*γ* response, and lymphocyte infiltration.(c) Scatter plot showing Spearman correlations between ERMP1 expression and the infiltration levels of various immune cell subsets in KIRC. ERMP1 is positively correlated with naive B cells and macrophages and shows distinct associations with T cell subsets.(d) Box plot comparing the tumor‐infiltrating lymphocyte (MeTIL) score between ERMP1 high‐ and low‐expression groups in KIRC (*p* = 0.004).(e) Comprehensive heat map illustrating the broad association between ERMP1 expression and the abundance of diverse immune cell subsets and cytotoxic activity.

**Figure:**
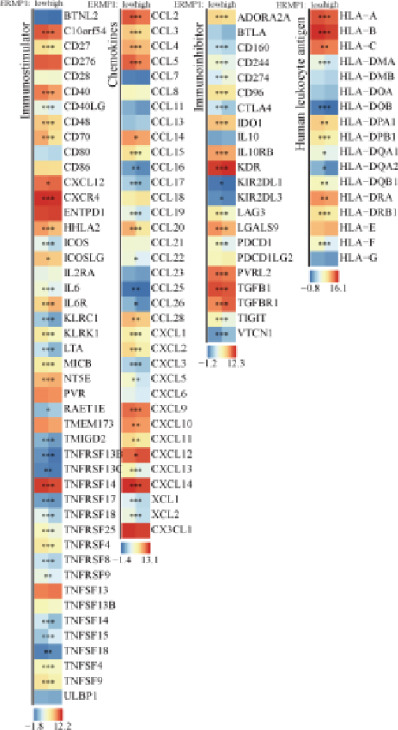
(a)

**Figure:**
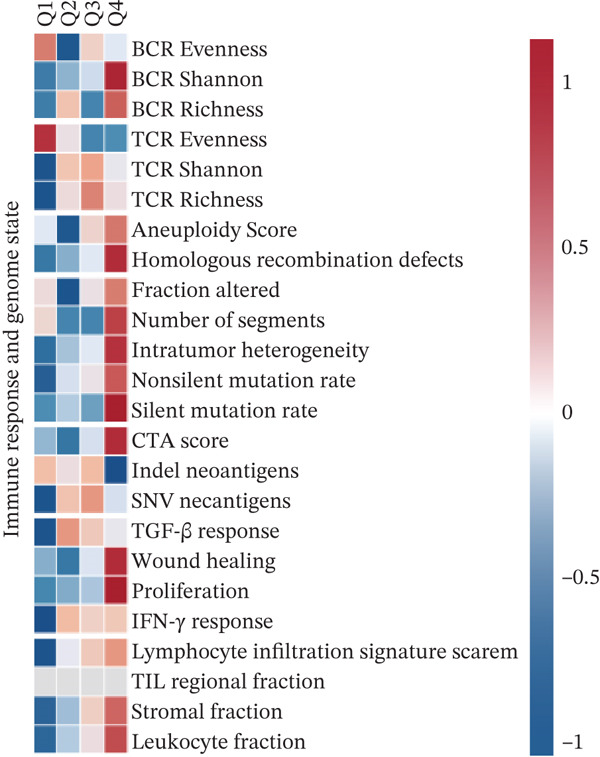
(b)

**Figure:**
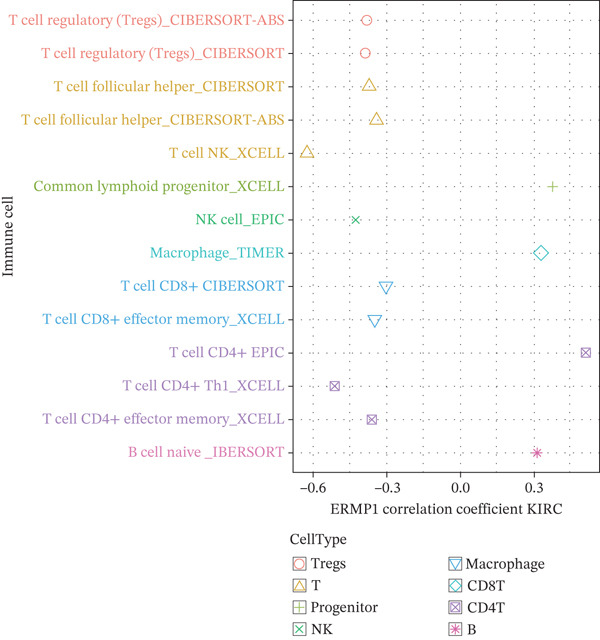
(c)

**Figure:**
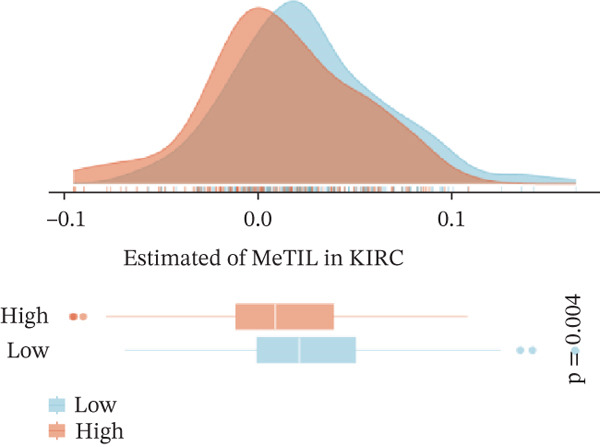
(d)

**Figure:**
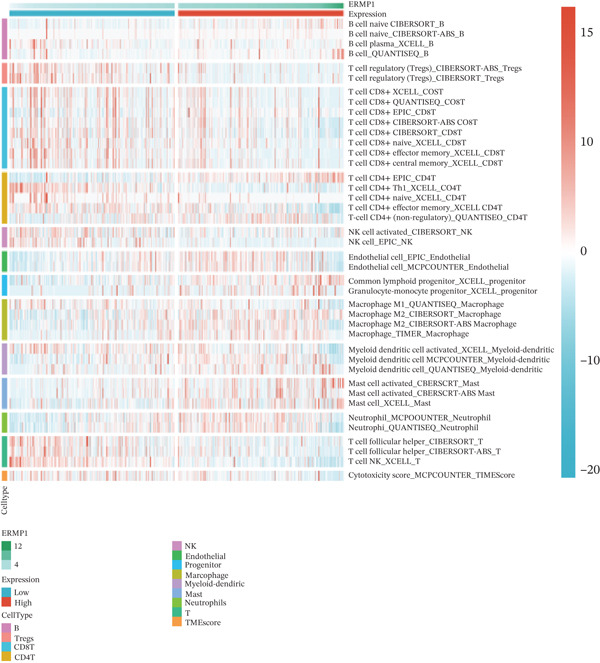
(e)

### 3.6. Analysis of Genetic Alterations, Expression Features, and Clinical Prognostic Value of ERMP1 in Cancer

To investigate the genetic alterations and potential functions of ERMP1 across cancers, we conducted a systematic analysis of a cohort comprising 370 tumor samples. Genetic variation analysis revealed an overall somatic mutation frequency of 3.24% for ERMP1, with its mutation spectrum encompassing various alteration types (Figure 6a). Functional enrichment analysis of ERMP1‐associated genes indicated significant enrichment in biological processes such as cell cycle regulation and metabolic pathways (Figure 6b), suggesting their potential collective involvement in ERMP1‐related carcinogenic mechanisms. To determine the differential expression of ERMP1 between tumor and adjacent normal tissues and its diagnostic potential, we performed expression and diagnostic efficacy analyses. Compared with paired adjacent normal tissues, ERMP1 expression was significantly downregulated in tumor tissues (Wilcoxon test, *p* < 0.001; Figure 6c,d). Notably, ERMP1 demonstrated excellent diagnostic performance in distinguishing tumor from normal tissue, achieving an area under the receiver operating characteristic (ROC) curve (AUC) of 0.963 (95% CI: 0.946–0.980; Figure 6e), indicating exceptionally high diagnostic potential. To evaluate the prognostic value of ERMP1 and construct a clinically applicable predictive tool, we performed systematic survival analysis and model construction. Survival analysis showed that high ERMP1 expression was significantly associated with longer OS (HR = 0.48, p < 0.001; Figure 6f) and DSS (HR = 0.40, *p* < 0.001; Figure 6g,h) in patients. Both univariate and multivariate Cox regression analyses confirmed ERMP1 as an independent protective prognostic factor in KIRC (multivariate: HR = 0.674, *p* = 0.018; Table 2). Based on this, we integrated ERMP1 expression with other clinical parameters to construct a nomogram for effectively predicting patients′ 3‐year and 5‐year survival probabilities (Figure 6i). The calibration curve of this model demonstrated high consistency between predicted outcomes and observed values (Figure 6j), confirming its robust predictive accuracy and potential clinical applicability.

Figure 6Genetic alterations, expression patterns, and clinical prognostic value of ERMP1 across human cancers. (a) Landscape of genetic alterations in ERMP1 across a pan‐cancer cohort (*n* = 370), including mutation frequency and variant types. Tumor mutation burden (TMB) is shown for each sample. (b) Ridge plot of functional enrichment analysis for ERMP1‐associated genes, showing significantly enriched pathways. Color scale indicates adjusted *p* values. (c) Scatter box plot comparing ERMP1 expression between tumor and adjacent normal tissues (Wilcoxon test). (d) Density distribution of ERMP1 expression in tumor versus normal tissues (Wilcoxon test). (e) Receiver operating characteristic (ROC) curve evaluating the diagnostic performance of ERMP1 for cancer detection. (f) Kaplan–Meier curve of overall survival (OS) stratified by ERMP1 expression level. (g) Kaplan–Meier curve of disease‐specific survival (DSS) based on ERMP1 expression. (h) Stratified Kaplan–Meier analysis of DSS for validation (HR = 0.40, 95% CI: 0.26–0.60). (i) Nomogram integrating ERMP1 expression and clinicopathological variables for predicting 3‐ and 5‐year survival probability. (j) Calibration curves of the nomogram for 3‐ and 5‐year survival prediction.

**Figure:**
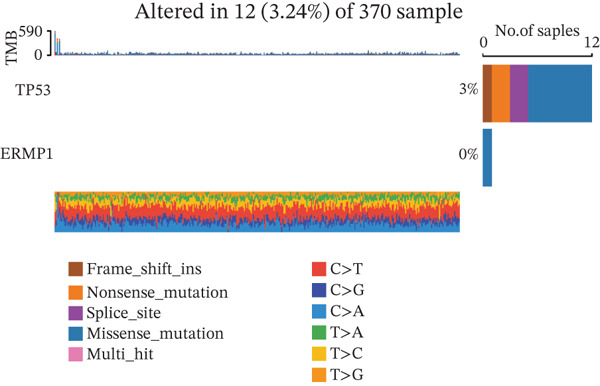
(a)

**Figure:**
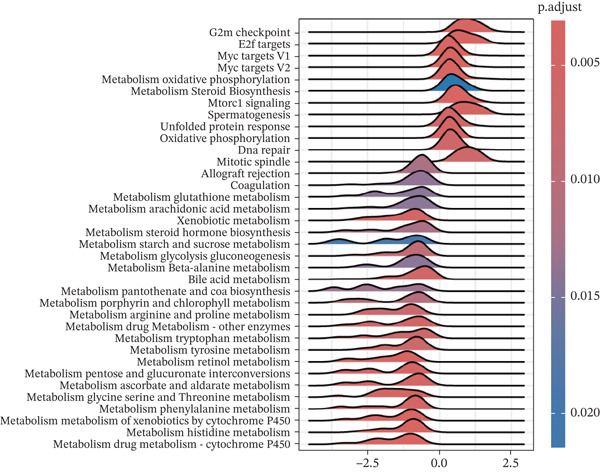
(b)

**Figure:**
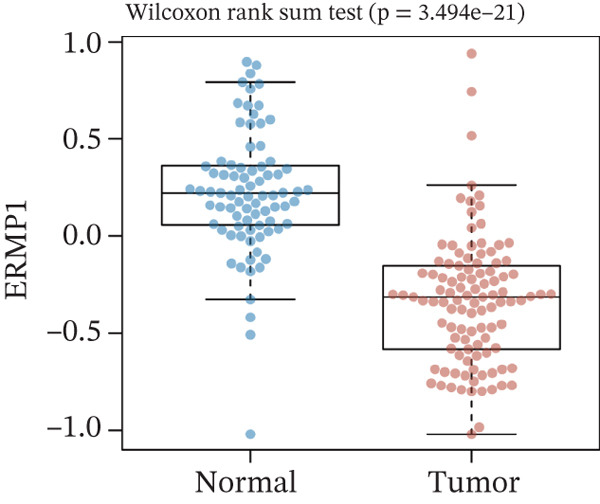
(c)

**Figure:**
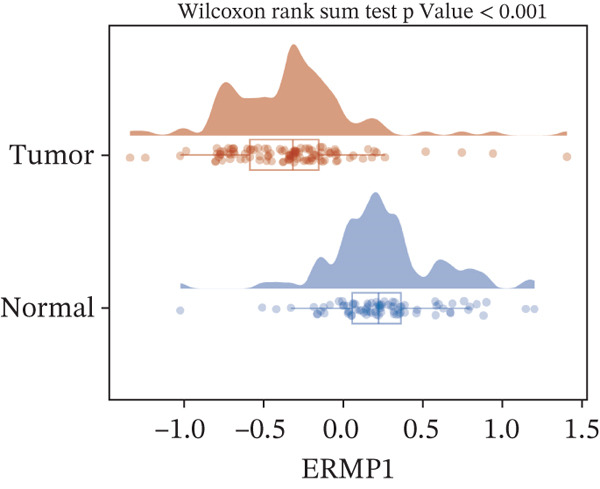
(d)

**Figure:**
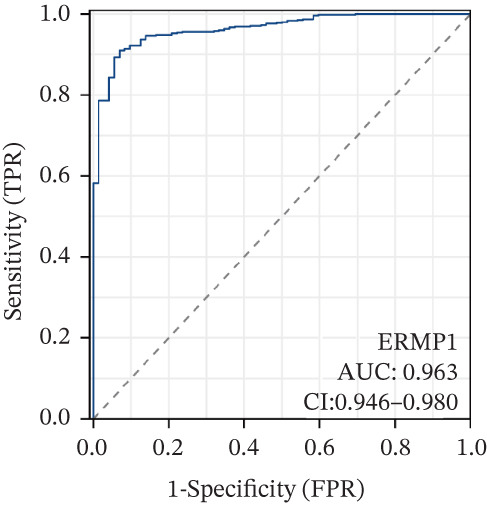
(e)

**Figure:**
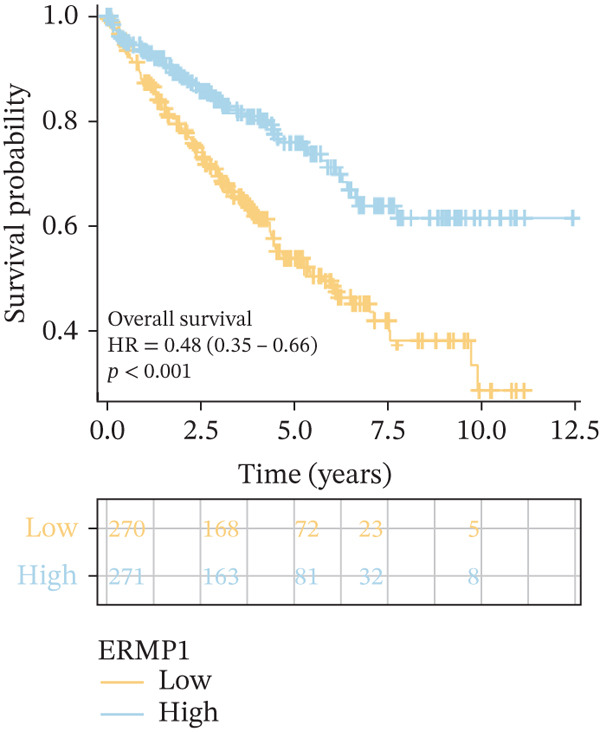
(f)

**Figure:**
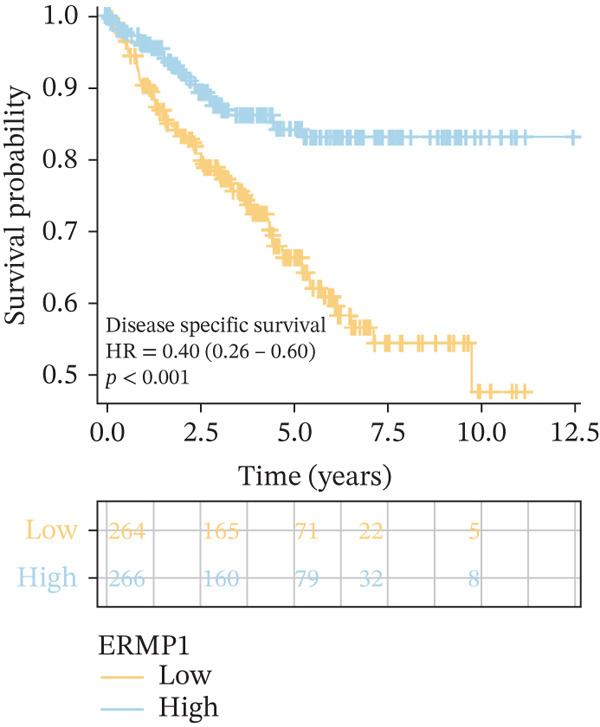
(g)

**Figure:**
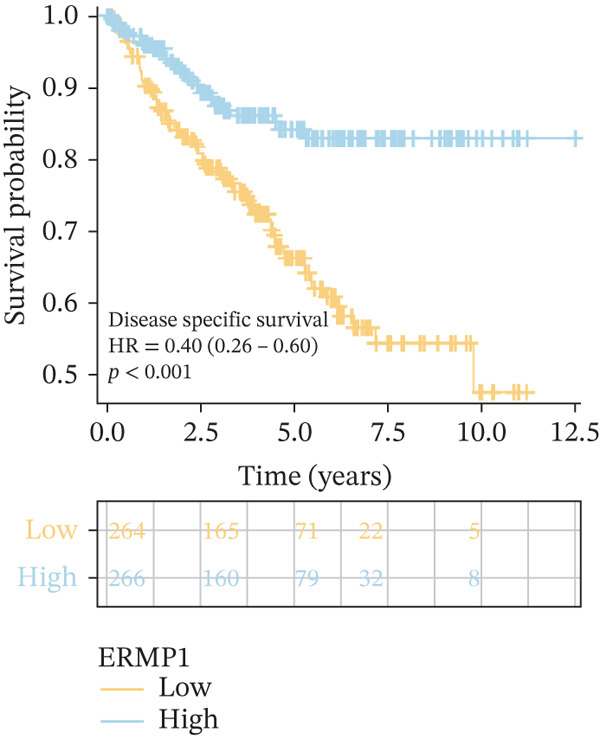
(h)

**Figure:**
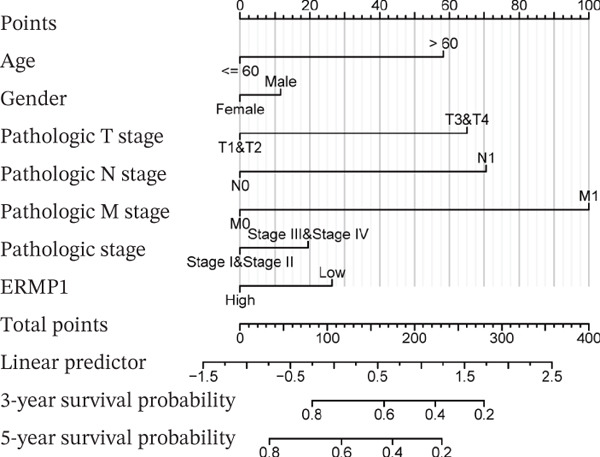
(i)

**Figure:**
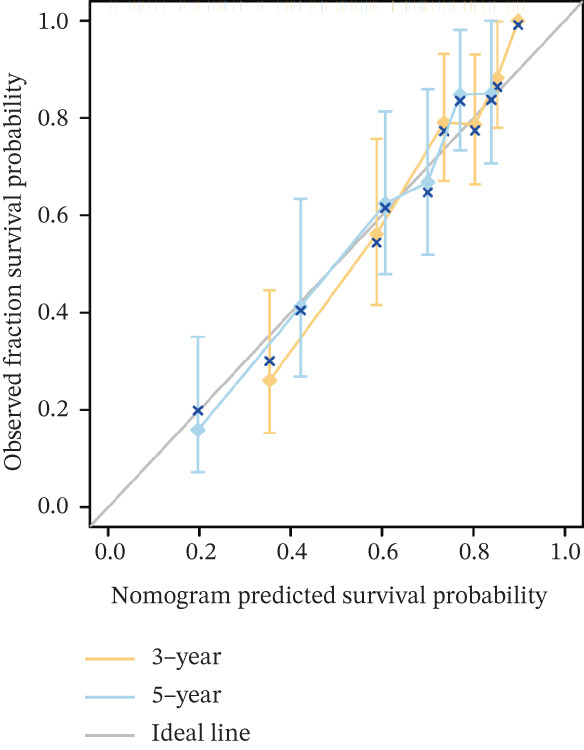
(j)

**Table 2:** Univariate and multivariate Cox regression analysis of the correlation between ERMP1 protein expression and clinicopathological characteristics in ccRCC patients.

Characteristics	Total (*N*)	Univariate analysis		Multivariate analysis	
		Hazard ratio (95% CI)	*p*	Hazard ratio (95% CI)	*p*
Age	541				
≤ 60	269	Reference		Reference	
> 60	272	1.791 (1.319–2.432)	**< 0.001**	1.743 (1.134–2.681)	**0.011**
Gender	541				
Female	187	Reference			
Male	354	0.924 (0.679–1.257)	0.613		
Pathologic *T* stage	541				
T1 and T2	350	Reference		Reference	
T3 and T4	191	3.210 (2.373–4.342)	**< 0.001**	1.941 (0.863–4.369)	0.109
Pathologic *N* stage	258				
N0	242	Reference		Reference	
N1	16	3.422 (1.817–6.446)	**< 0.001**	1.979 (1.017–3.851)	**0.044**
Pathologic M stage	508				
M0	429	Reference		Reference	
M1	79	4.401 (3.226–6.002)	**< 0.001**	2.712 (1.622–4.534)	**< 0.001**
Pathologic stage	538				
Stage I and Stage II	332	Reference		Reference	
Stage III and Stage IV	206	3.910 (2.852–5.360)	**< 0.001**	1.205 (0.479–3.034)	0.692
ERMP1	541				
Low	270	Reference		Reference	
High	271	0.477 (0.348–0.655)	**< 0.001**	0.674 (0.486–0.935)	**0.018**

*Note:* The bolded values denote statistically significant results (e.g., values meeting the predefined significance threshold of *p* < 0.05, *p* < 0.01, or other established statistical criteria).

### 3.7. ERMP1 Is Downregulated in Renal Cell Carcinoma and Its Overexpression Suppresses Malignant Phenotypes

To investigate the expression pattern and function of ERMP1 in KIRC, we conducted a systematic analysis at both tissue and cellular levels. Immunohistochemical staining showed that ERMP1 protein expression was significantly lower in KIRC tissues than in normal tissues (Figure 7a). Western blot analysis further confirmed that ERMP1 protein levels were markedly reduced in tumor tissues from multiple patient samples compared with matched adjacent normal tissues (Figure 7b,c). Consistent with these findings, qPCR analysis indicated that ERMP1 mRNA expression was also significantly downregulated in tumor tissues (Figure 7f). To elucidate the biological role of ERMP1, we established an ERMP1 overexpression model in the Caki‐1 and A498 KIRC cell lines. Both Western blot and qPCR results confirmed efficient overexpression of ERMP1 at the protein and mRNA levels after transfection (Figure 7d,e). GFP fluorescence observation verified good transfection efficiency (Figure 7i). Functional assays demonstrated that overexpression of ERMP1 significantly suppressed malignant phenotypes of KIRC cells: Transwell assays showed a significant reduction in the number of invading cells after ERMP1 overexpression (Figure 7g,h); wound healing assays indicated that ERMP1 markedly delayed the wound closure rate (Figure 7k,l); colony formation assays revealed that both the number and size of colonies were significantly smaller in the ERMP1 overexpression group compared with the control group (Figure 7j,m). Furthermore, CCK‐8 proliferation assays confirmed that ERMP1 overexpression significantly inhibited cell proliferation activity (Figure 7n). In summary, these results indicated that ERMP1 is downregulated in renal cell carcinoma, and restoring its expression effectively suppresses malignant phenotypes of tumor cells.

Figure 7Elevated expression of ERMP1 in KIRC and functional characterization of its tumor‐suppressive roles.(a) Immunohistochemical staining of ERMP1 in KIRC tissues. (b, c) Western blot analysis (b) and quantitative results (c) of ERMP1 protein expression in paired normal (N) and tumor (T) tissues from KIRC patients. GAPDH was used as a loading control. (D, E) Efficiency of ERMP1 overexpression verified by Western blot (d) and quantitative real‐time PCR (e) in Caki‐1 and A498 cells. GAPDH was used as a loading control for Western blot. (f) Quantitative real‐time PCR analysis of ERMP1 mRNA expression in normal and KIRC tissues. (g, h) Transwell invasion assays showing invaded cells (g) and quantitative analysis (h) of Caki‐1 and A498 cells after ERMP1 overexpression. (i) GFP fluorescence imaging showing transfection efficiency in Caki‐1 and A498 cells. (j, m) Colony formation assays showing representative images (j) and quantitative analysis (m) of Caki‐1 and A498 cells after ERMP1 overexpression. (k, l) Wound healing assays showing migration at 0 and 24 hours (k) and quantitative analysis of wound closure rate (l) in Caki‐1 and A498 cells.(N) CCK‐8 assays showing proliferation of Caki‐1 and A498 cells after ERMP1 overexpression. *n* = 3, all quantitative data are presented as mean ± SD.  ^∗^ *p* < 0.05,  ^∗∗^ *p* < 0.01,  ^∗∗^ *p* < 0.001 versus control group.

**Figure:**
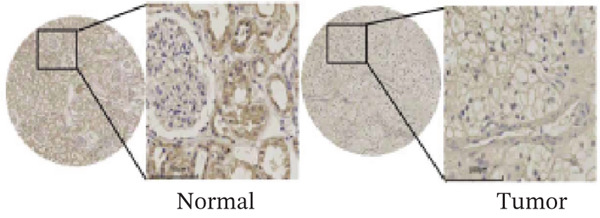
(a)

**Figure:**
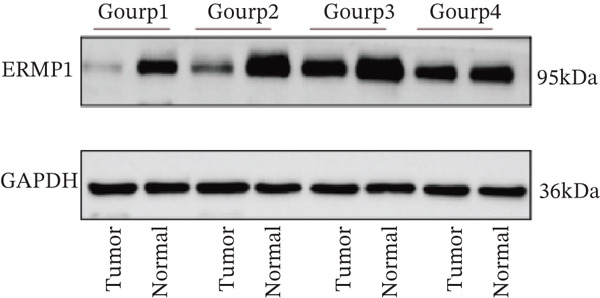
(b)

**Figure:**
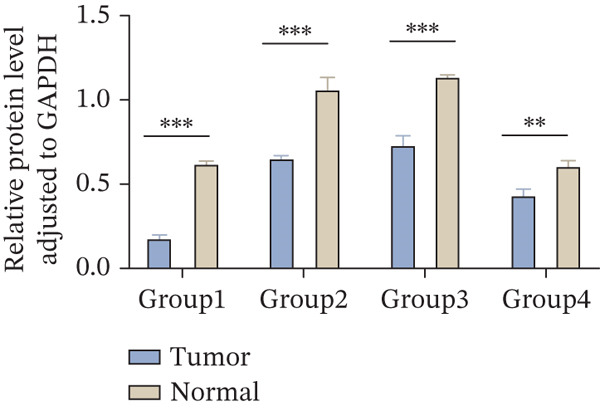
(c)

**Figure:**
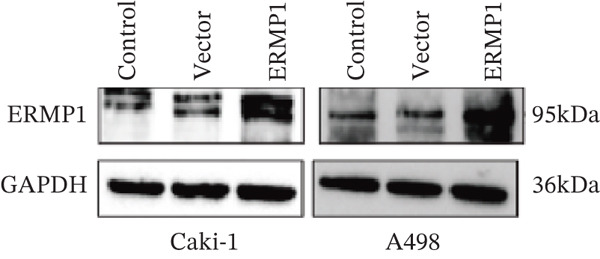
(d)

**Figure:**
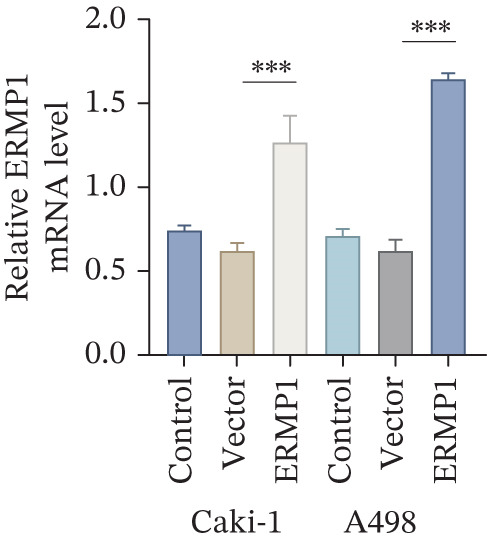
(e)

**Figure:**
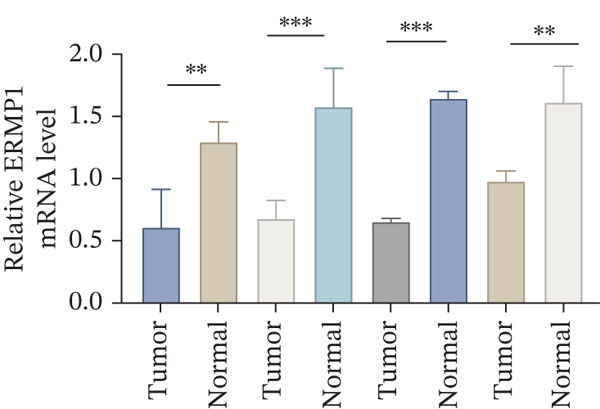
(f)

**Figure:**
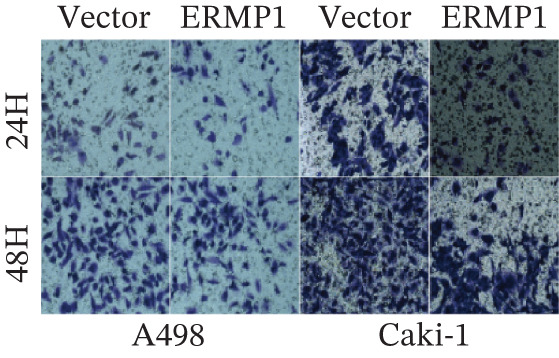
(g)

**Figure:**
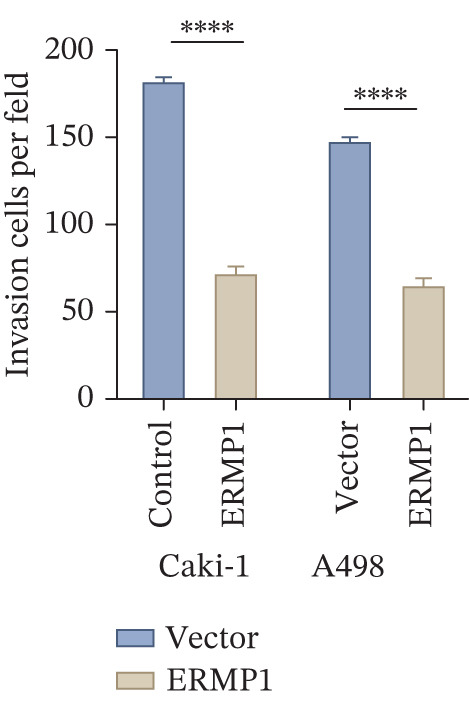
(h)

**Figure:**
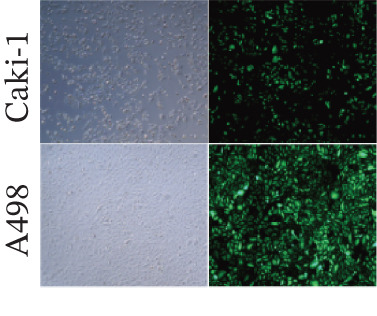
(i)

**Figure:**
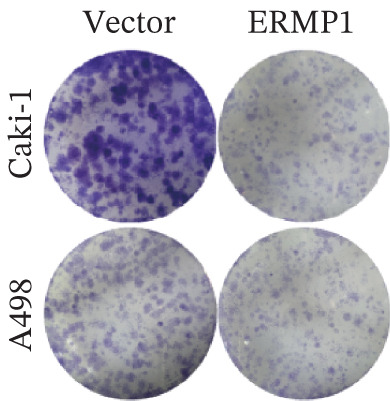
(j)

**Figure:**
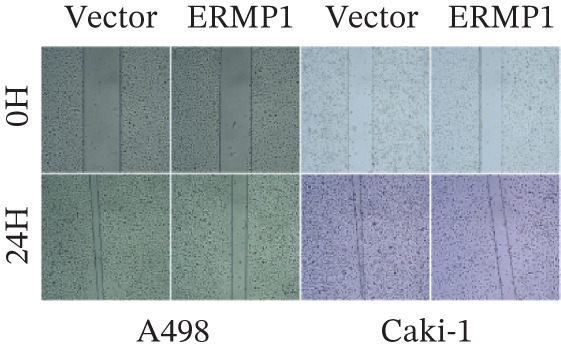
(k)

**Figure:**
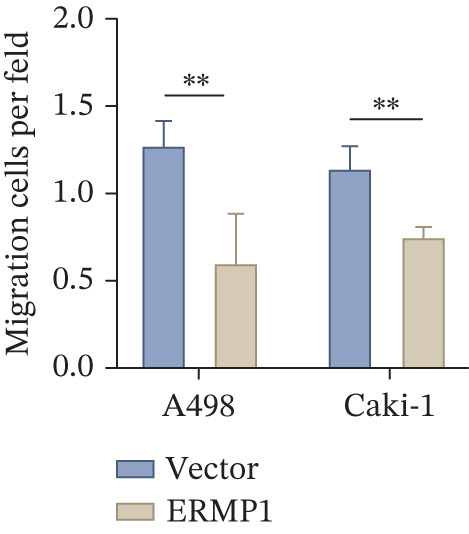
(l)

**Figure:**
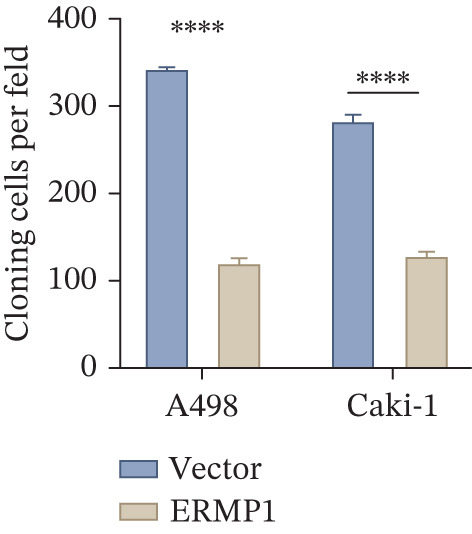
(m)

**Figure:**
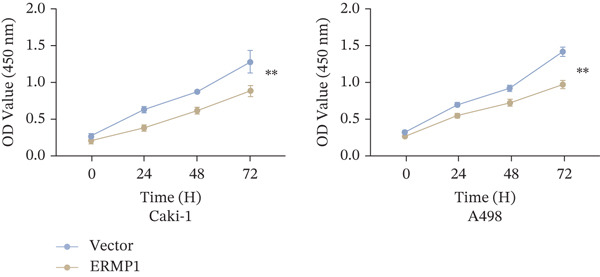
(n)

### 3.8. ERMP1 Suppresses Tumor Progression by Inhibiting the PI3K/AKT Signaling Pathway

To systematically elucidate the functional mechanisms of ERMP1, we employed an integrated approach combining bioinformatic prediction with experimental validation. Pan‐cancer pathway activity analysis revealed that ERMP1 exerts cancer‐type–specific regulatory effects on fundamental processes including apoptosis and cell cycle (Figure 8a). Chord diagram analysis further demonstrated significant interactions between ERMP1 and core oncogenic pathways, with particularly strong associations with PI3K/AKT signaling, EMT, and cell cycle regulation (Figure 8b). Based on these findings, we focused on validating ERMP1′s role in PI3K/AKT signaling. Western blot analysis demonstrated that ERMP1 overexpression significantly suppressed the phosphorylation of key pathway components PI3K and AKT in both Caki‐1 and A498 renal cancer cell lines, without affecting their total protein levels (Figure 8c). Densitometric quantification confirmed these observations, showing remarkable reduction in p‐PI3K and p‐AKT levels following ERMP1 overexpression (Figure 8e,f). We next investigated downstream functional effects of PI3K/AKT pathway inhibition by ERMP1. Western blot analysis showed that ERMP1 overexpression markedly reversed EMT, as evidenced by increased E‐cadherin expression and decreased N‐cadherin and vimentin levels (Figure 8d). Concurrently, ERMP1 suppressed the expression of invasion‐related matrix metalloproteinases (MMP2 and MMP9) and cell cycle regulators (Cyclin D1 and CDK4). Quantitative analysis validated these protein expression changes in both cell lines (Figure 8g,h). To verify the tumor‐suppressive effect of ERMP1 in vivo, we established a nude mouse xenograft tumor model using A498 cells with stable ERMP1 overexpression. The results showed that, compared with the control group, the tumor growth rate of mice in the ERMP1 overexpression group was significantly reduced, and the final tumor weight was also markedly decreased (Figure 8i,j). These results demonstrate that ERMP1 downregulates malignant biological behaviors including EMT progression, cell invasive capacity and cell cycle progression by inhibiting the PI3K/AKT signaling pathway, and further confirms that ERMP1 can significantly suppress tumor growth in an in vivo xenograft tumor model.

Figure 8ERMP1 inhibits tumor progression by suppressing the PI3K/AKT signaling pathway. (a) Heatmap depicting pathway regulation by ERMP1 across multiple cancer types. Color intensity represents regulatory activity of ERMP1 on various pathways (red: activation; blue: inhibition). (b) Chord diagram illustrating interactions between ERMP1 and core signaling pathways. Ribbon width indicates interaction strength, whereas color represents regulatory direction (red: positive correlation; green: negative correlation). (c) Western blot analysis of phosphorylated protein levels in the PI3K/AKT pathway following ERMP1 overexpression. GAPDH was used as loading control. (d) Western blot analysis of EMT, invasion, and cell cycle‐related proteins after ERMP1 overexpression. GAPDH was used as loading control. (e, f) Quantitative analysis of key PI3K/AKT pathway proteins in (e) Caki‐1 and (f) A498 cells, *n* = 3, data are presented as mean ± SD,  ^∗∗∗^p < 0.001; ns, not significant. (g, h) Quantitative analysis of downstream functional proteins in (g) Caki‐1 and (h) A498 cells, *n* = 3, data are presented as mean ± SD,  ^∗∗∗^p < 0.001. (i–j) In vivo xenograft tumor assay: Tumor tissue photographs of mice in the Vector and ERMP1 groups, as well as the quantitative statistical results of tumor weights (*n* = 4), data are presented as *m* *e* *a* *n* ± *S* *D*,  ^∗∗^ *p* < 0.01.

**Figure:**
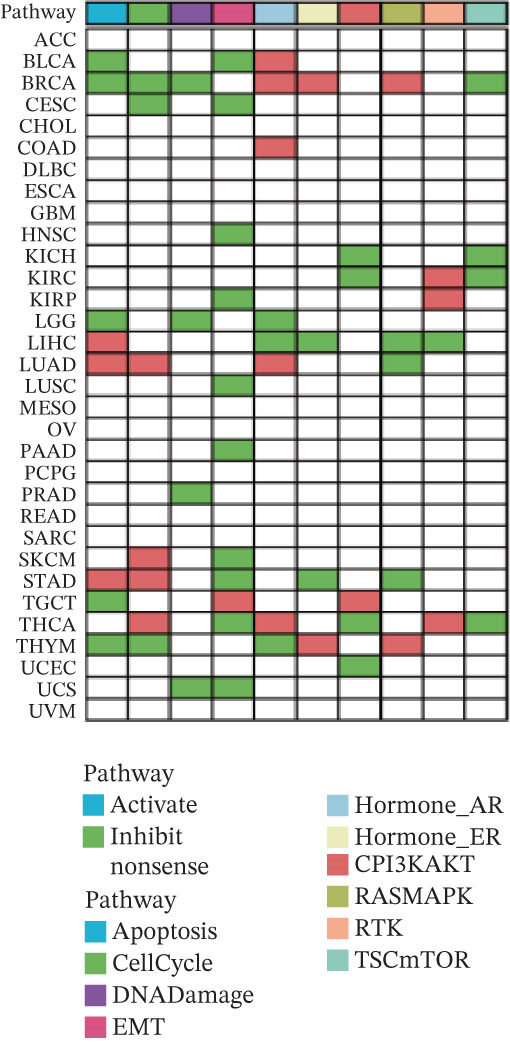
(a)

**Figure:**
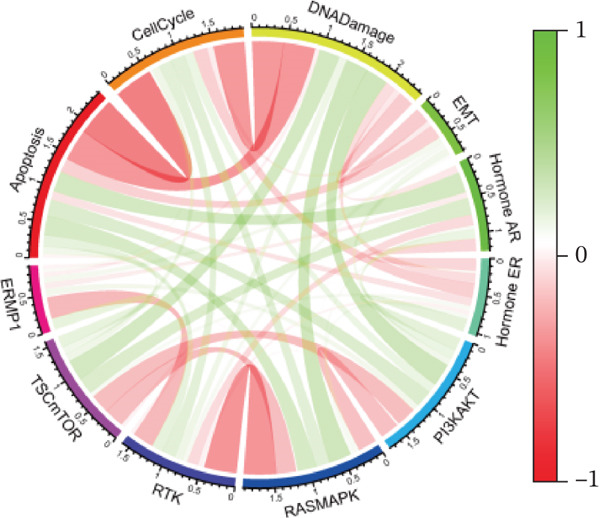
(b)

**Figure:**
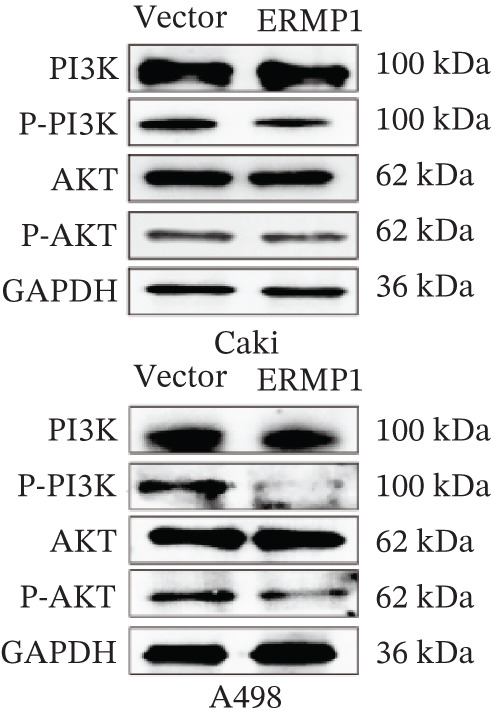
(c)

**Figure:**
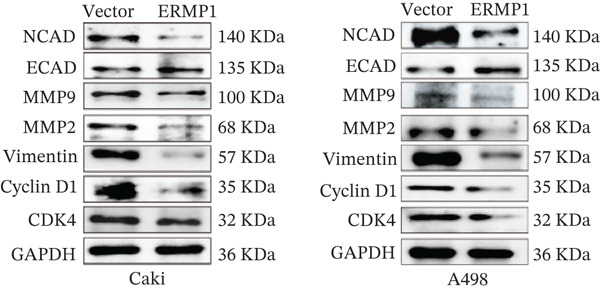
(d)

**Figure:**
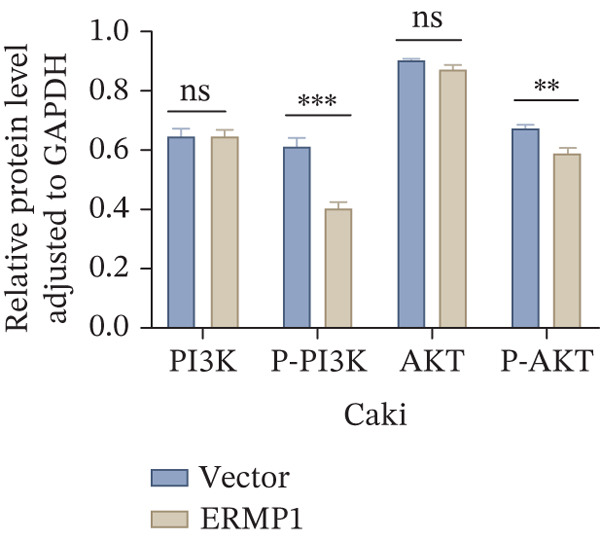
(e)

**Figure:**
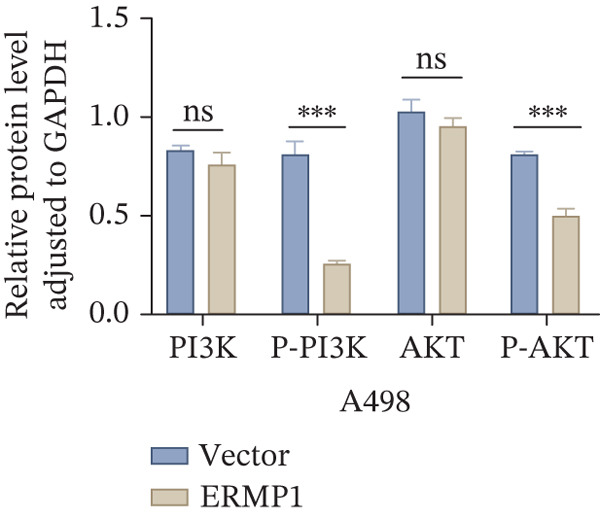
(f)

**Figure:**
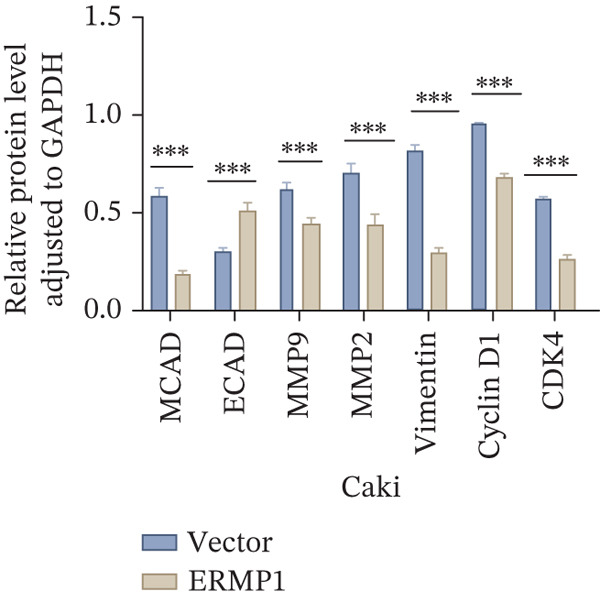
(g)

**Figure:**
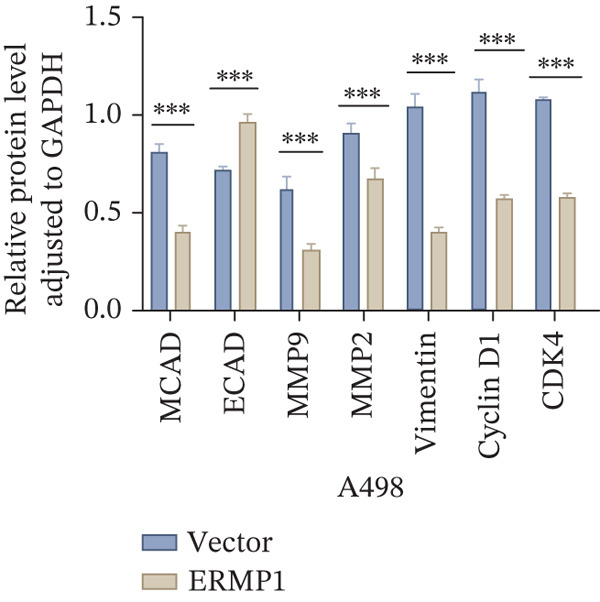
(h)

**Figure:**
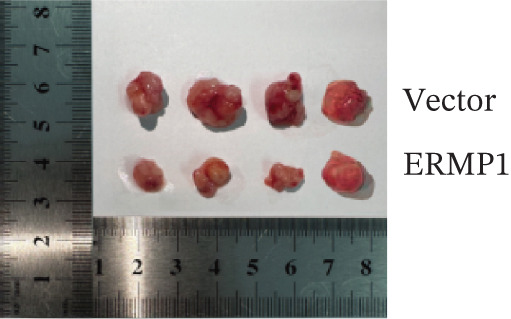
(i)

**Figure:**
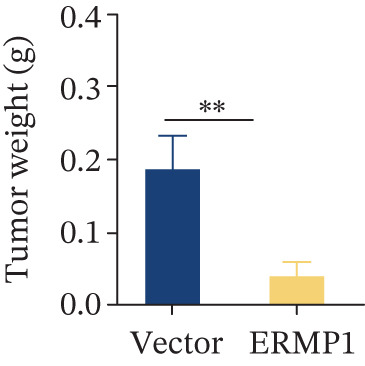
(j)

## 4. Discussion

This study integrates pan‐cancer analysis with functional experiments to systematically demonstrate that ERMP1, a key regulator of ER homeostasis, exhibits remarkable cancer‐type specificity in its expression patterns, prognostic significance, and immunomodulatory roles. By combining multiomics data and in vitro validation, we confirmed that ERMP1 acts as a tumor suppressor in KIRC, significantly inhibiting tumor cell proliferation, migration, invasion, and clonogenic ability. Mechanistically, ERMP1 exerts its tumor‐suppressive function by negatively regulating the PI3K/AKT signaling pathway, thereby suppressing epithelial‐mesenchymal transition (EMT), downregulating the expression of invasion‐related proteins and inducing cell cycle arrest. Furthermore, it has been confirmed in in vivo xenograft tumor models that ERMP1 overexpression can significantly inhibit tumor growth. Our findings elucidate the functional heterogeneity of ERMP1 across different cancers, clarify its tumor‐suppressive role in KIRC, and provide a theoretical foundation for developing targeted therapeutic strategies.

Previous studies have implicated ERMP1 in coordinating multiple stress adaptation mechanisms—including ER stress, oxidative stress, and hypoxia response—to promote tumor cell survival, highlighting its potential as a target for overcoming treatment resistance [12, 23, 24]. Our pan‐cancer analysis provides the first systematic overview of ERMP1 expression across tissues, revealing significant upregulation in digestive system malignancies such as COAD and esophageal carcinoma (ESCA). Interestingly, ERMP1 exhibits low expression in KIRC tissues, and its low expression is associated with poor prognosis in patients, indicating a unique, context‐dependent tumor‐suppressive role in this malignancy. Further genetic characterization identified STAD as having the highest frequency of ERMP1 alterations, primarily driven by copy number deletions (*p* < 0.001), suggesting that genomic instability may be an important regulator of its expression. The tissue‐specific influence of ERMP1 is even more evident in prognostic associations. While Zhao Yong et al. identified high ERMP1 expression as a risk factor in osteosarcoma [22], both our study and recent literature confirm its role as an independent favorable prognostic factor in KIRC [18]. Pan‐cancer analysis further supported this, linking high ERMP1 expression to improved DSS and PFI in KIRC patients. Moreover, ERMP1 exhibited high diagnostic accuracy (AUC = 0.963) in distinguishing KIRC tumor from normal tissue, surpassing conventional clinical markers [1, 25]. Together, these findings underscore the potential of ERMP1 as an independent prognostic biomarker in KIRC.

ERMP1 contributes to proteostatic maintenance and supports antitumor immunity through its regulation of ER stress. However, dysregulation of ERMP1 and subsequent chronic activation of the UPR may facilitate tumor progression and immune evasion [23, 26]. In osteosarcoma, high ERMP1 expression promotes an immunosuppressive TME by reducing antitumor T cell infiltration and expanding immunosuppressive cell populations, thereby accelerating tumor progression [18]. Our immune cell expression profiling revealed elevated ERMP1 expression in Tregs and Tprolif, implying a direct role in immune cell modulation. In KIRC, high ERMP1 expression is associated with an immunologically active TME, marked by upregulation of immune stimulators (CD40 and CD80), chemokines, and HLA molecules, increased BCR/TCR diversity, enhanced IFN‐*γ* response, and elevated overall lymphocytic infiltration (MeTIL score). ERMP1 expression also positively correlates with infiltration of naïve B cells and macrophages, though its association with certain T cell subsets is more variable. These observations suggest that in KIRC, ERMP1 may foster an antitumor immune milieu by enhancing antigen presentation, promoting immune diversity, and facilitating lymphocyte recruitment—offering a novel perspective on its tumor‐suppressive mechanism.

Prior studies have highlighted the tissue‐specific oncogenic roles of ERMP1. In colorectal cancer, it drives proliferation through PI3K/AKT pathway activation11; in endometrial cancer, it upregulates the HIF‐1/Nrf2 axis to enhance stress tolerance [24]; and in nonsmall cell lung cancer, it primarily promotes cell proliferation [27, 28]. Of note, ERMP1 is broadly expressed across renal cell types and is upregulated in chronic kidney disease, likely as a compensatory response to ER stress [4, 25, 29]. Nonetheless, its functional significance in KIRC remained poorly defined. Here, using in vitro models, we provide the first experimental evidence supporting a tumor‐suppressive role for ERMP1 in KIRC. Overexpression of ERMP1 significantly impaired the proliferative, migratory, and invasive abilities of Caki‐1 and A498 cells. At the molecular level, ERMP1 reversed EMT by upregulating E‐cadherin and downregulating N‐cadherin and vimentin. GSEA and Western blot analyses further indicated that these effects are mediated through inhibition of the PI3K/AKT pathway. Clinical corroboration came from multivariate Cox regression, which confirmed ERMP1 as an independent protective prognostic factor in KIRC patients. A nomogram incorporating ERMP1 demonstrated strong calibration and discriminative ability in predicting 3‐ and 5‐year survival, supporting its potential clinical applicability. Notably, ERMP1 is not a classical component of the PI3K/AKT pathway, and its effects on tumor cell signal transduction and immune‐related phenotypes are likely mediated through indirect mechanisms. Building on prior research, ERMP1‐mediated regulation of ER stress/UPR may serve as a potential mechanistic link between its intracellular tumor‐suppressive function and the TIME by reprogramming the immune‐related secretory profile of tumor cells.

Several limitations of this study should be acknowledged. First, the limited sample size for certain cancer types (particularly rare cancers) may constrain the statistical robustness of some pan‐cancer conclusions. Second, current experimental evidence is primarily based on ERMP1 overexpression models. Our Western blot results indicate that elevated ERMP1 expression correlates with reduced activity of the PI3K/AKT pathway; however, these data alone are insufficient to demonstrate that ERMP1 acts as a direct upstream negative regulator of this pathway. The specific molecular mechanisms underlying their mutual regulation remain to be further elucidated in subsequent studies. Finally, the in vitro findings presented here warrant further validation using in vivo models, such as KIRC xenograft studies, to more comprehensively assess the functional impact of ERMP1 in tumor development and progression.

In conclusion, this work systematically outlines the expression patterns, prognostic relevance, and immune‐related functions of ERMP1 across cancer types. It also offers the first experimental evidence that ERMP1 exerts tumor‐suppressive activity in KIRC via inhibition of the PI3K/AKT pathway. These insights advance our understanding of ERMP1′s functional heterogeneity and establish a foundation for its future development as a diagnostic marker, prognostic indicator, or therapeutic target in KIRC.

## 5. Conclusion

Combining systematic pan‐cancer analysis with functional validation, this study reveals the tissue‐specific roles of ERMP1 in cancer. ERMP1 exhibits tumor‐promoting effects in digestive system malignancies such as COAD and ESCA, while acting as a tumor suppressor in KIRC. In KIRC, low ERMP1 expression correlates with poor prognosis, whereas its overexpression suppresses tumor cell proliferation, migration, invasion, EMT, and induces cell cycle arrest by inhibiting the PI3K/AKT pathway. Moreover, high ERMP1 expression is associated with an immunologically active TME and enhanced antitumor immunity. These findings elucidate the context‐dependent functions of ERMP1 and support its potential as a prognostic biomarker and therapeutic target in KIRC.

**Nomenclature**
ACC: adrenocortical carcinoma
BLCA: bladder urothelial carcinoma
BRCA: breast invasive carcinoma
CESC: cervical squamous cell carcinoma
CHOL: cholangiocarcinoma
COAD: colon adenocarcinoma
ESCA: esophageal carcinoma
GBM: glioblastoma multiforme
HNSC: head and neck squamous cell carcinoma
KICH: kidney chromophobe
KIRC: kidney renal clear cell carcinoma
KIRP: kidney renal papillary cell carcinoma
LAML: acute myeloid leukemia
LGG: brain lower grade glioma
LIHC: liver hepatocellular carcinoma
LUAD: lung adenocarcinoma
LUSC: lung squamous cell carcinoma
OV: ovarian serous cystadenocarcinoma
MESO: mesothelioma
PAAD: pancreatic adenocarcinoma
PCPG: pheochromocytoma and paraganglioma
PRAD: prostate adenocarcinoma
READ: rectum adenocarcinoma
SARC: sarcoma
SKCM: skin cutaneous melanoma
STAD: stomach adenocarcinoma
TGCT: testicular germ cell tumors
THCA: thyroid carcinoma
THYM: thymoma
UCEC: uterine corpus endometrial carcinoma
UCS: uterine carcinosarcoma
UVM: uveal melanoma

## Author Contributions

Ziyang Liu and Yang Feilong: conceptualization, study design. Jiahao Shan, Tao Yang, and Qiang Zhang: literature collection. Ziyang Liu and Jiahao Shan: writing—original draft, figure preparation, and manuscript editing. Feilong Yang and Lianghong Ma: critical revision of the manuscript. Ziyang Liu and Jiahao Shan contributed equally to this work.

## Funding

This study was supported by Natural Science Foundation of Ningxia Hui Autonomous Region (2022AAC05054).

## Disclosure

All authors read and approved the final manuscript.

## Ethics Statement

The acquisition and use of all human tissue samples in this study were strictly conducted in accordance with the principles of the Declaration of Helsinki. The research protocol was approved by the Research Ethics Committee of the General Hospital of Ningxia Medical University (Approval No. K021/2021). A total of 60 clear cell renal cell carcinoma (ccRCC) tissues and 60 matched nontumorous kidney specimens were collected from patients who underwent surgery at the General Hospital of Ningxia Medical University. All patients had complete clinical records and follow‐up data and had not received any form of tumor‐specific therapy before diagnosis.

## Conflicts of Interest

The authors declare no conflicts of interest.

## Supporting information


**Supporting Information** Additional supporting information can be found online in the Supporting Information section. Figure S1;The diagnostic efficacy of ERMP1 exhibits significant heterogeneity across different cancer types. Receiver operating characteristic (ROC) curves for discriminating tumor from normal tissues in 16 cancer types (a–p): (a) breast cancer (BRCA); (b) bladder urothelial carcinoma (BLCA); (c) cervical squamous cell carcinoma (CESC); (d) cholangiocarcinoma (CHOL); (e) colorectal cancer (COAD); (f) esophageal cancer (ESCA); (g) head and neck squamous cell carcinoma (HNSC); (h) hepatocellular carcinoma (LIHC); (i) lung squamous cell carcinoma (LUSC); (j) pancreatic cancer (PAAD); (k) prostate cancer (PRAD); (l) stomach adenocarcinoma (STAD); (m) thyroid cancer (THCA); (n) thymoma (THYM); (o) uterine corpus endometrial carcinoma (UCEC); and (p) oral squamous cell carcinoma (OSCC). Figure S2: Cancer‐type specific associations between ERMP1 and the tumor microenvironment. Correlation analysis of ERMP1 expression levels with tumor microenvironment components (including tumor purity, B cells, CD8^+^ T cells, CD4^+^ T cells, and macrophages) across multiple cancer types: breast cancer (BRCA), head and neck squamous cell carcinoma (HNSC), kidney renal clear cell carcinoma (KIRC), hepatocellular carcinoma (LIHC), pancreatic adenocarcinoma (PAAD), prostate adenocarcinoma (PRAD), and thyroid carcinoma (THCA).

## Data Availability

The original contributions presented in the study are included in the article. The datasets generated and/or analyzed during the current study are available from the corresponding authors on reasonable request.
