# Supplementary figures and images for: ERMP1 Exerts Tumor‐Suppressive Functions in KIRC by Inhibiting PI3K/AKT Signaling and Remodeling the Immune Microenvironment: A Pan‐Cancer Analysis
Source: Hum Mutat. 2026 Apr 7;2026:7717815. doi: 10.1155/humu/7717815 (PMC13057433; doi:10.1155/humu/7717815)

Supplementary Figure1


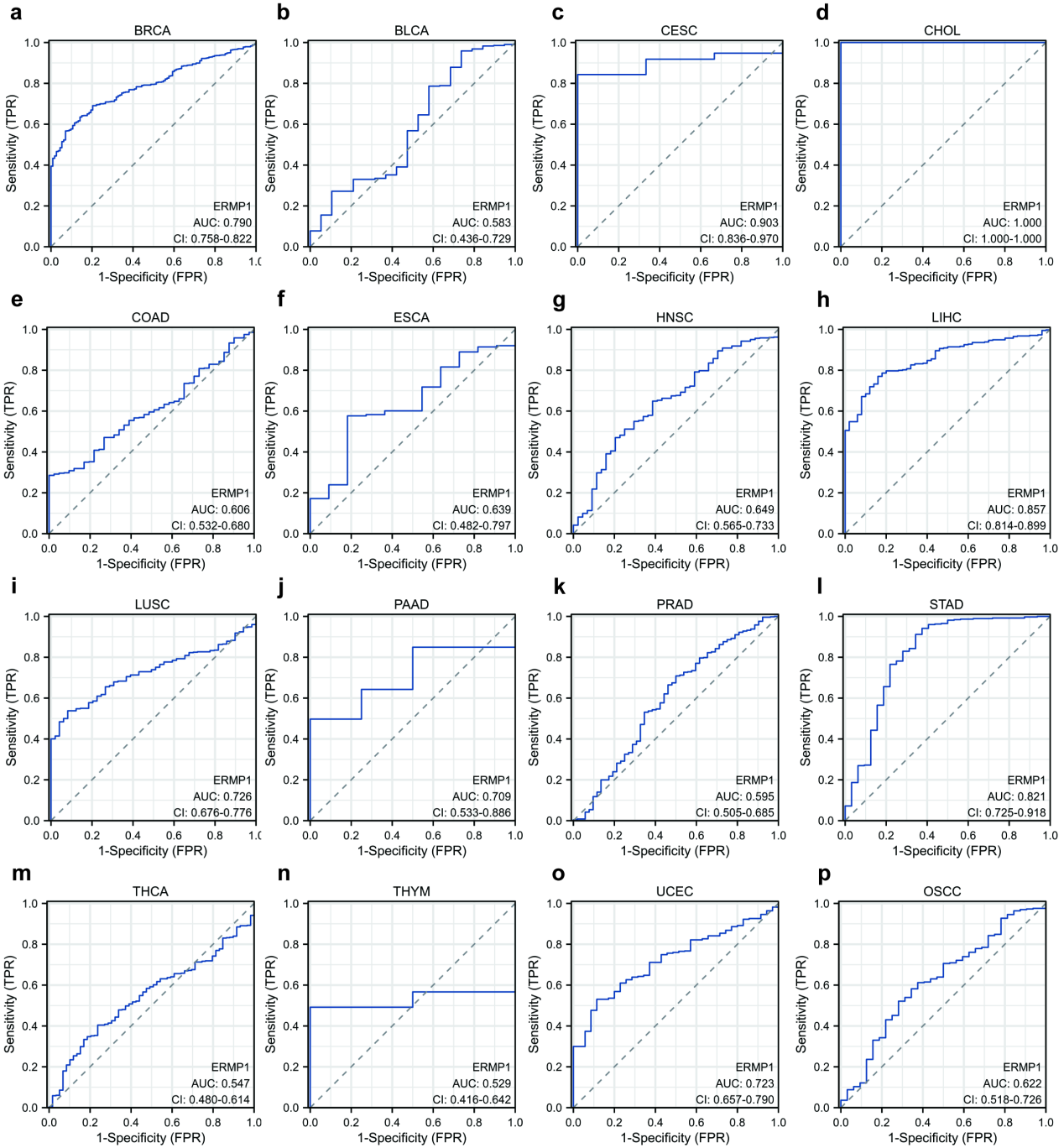


Supplementary Figure2


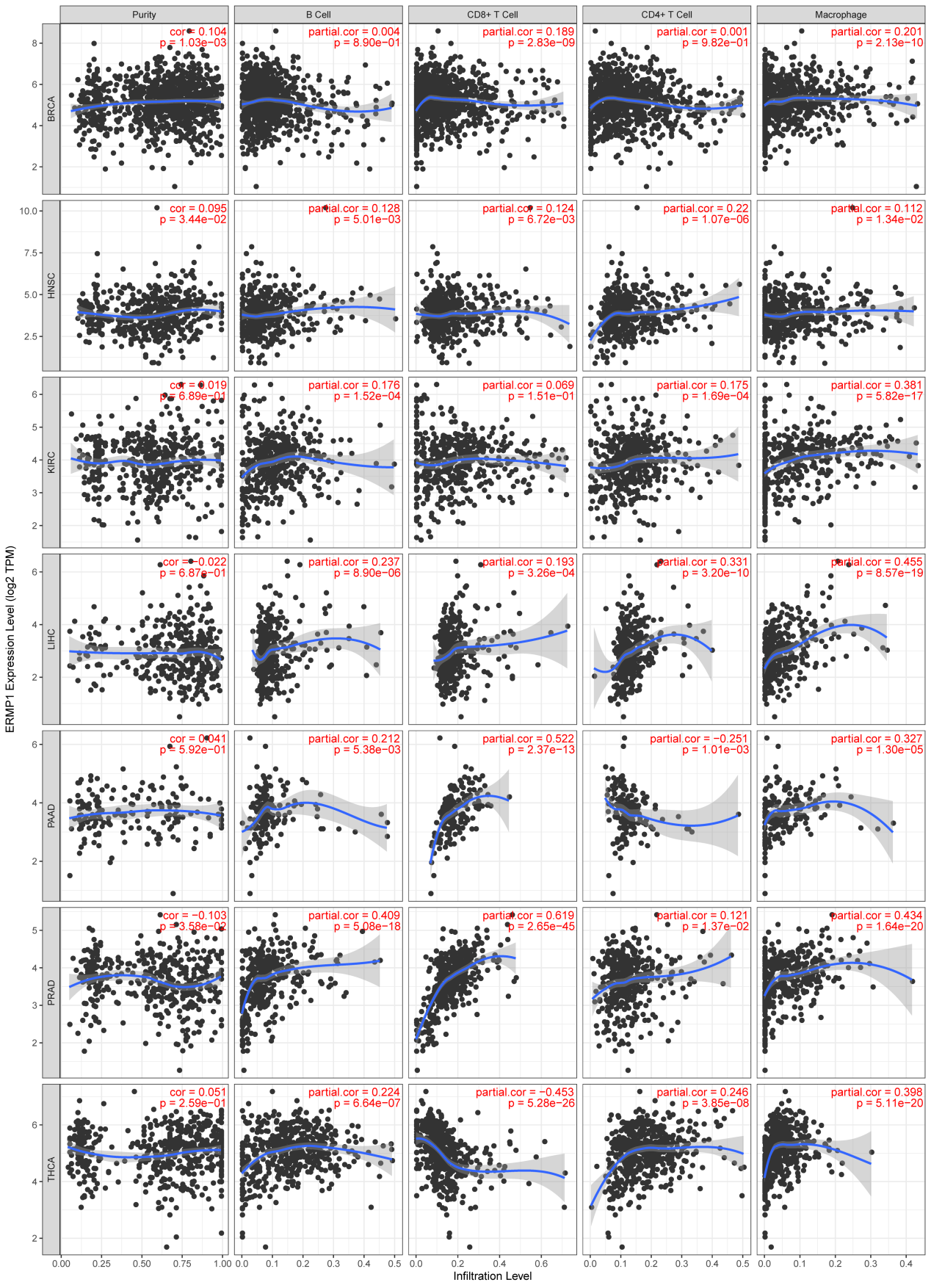

Supplement: Supplementary file 1 — Supporting Information Additional supporting information can be found online in the Supporting Information section. Figure S1;The diagnostic efficacy of ERMP1 exhibits significant heterogeneity across different cancer types. Receiver operating characteristic (ROC) curves for discriminating tumor from normal tissues in 16 cancer types (a–p): (a) breast cancer (BRCA); (b) bladder urothelial carcinoma (BLCA); (c) cervical squamous cell carcinoma (CESC); (d) cholangiocarcinoma (CHOL); (e) colorectal cancer (COAD); (f) esophageal cancer (ESCA); (g) head and neck squamous cell carcinoma (HNSC); (h) hepatocellular carcinoma (LIHC); (i) lung squamous cell carcinoma (LUSC); (j) pancreatic cancer (PAAD); (k) prostate cancer (PRAD); (l) stomach adenocarcinoma (STAD); (m) thyroid cancer (THCA); (n) thymoma (THYM); (o) uterine corpus endometrial carcinoma (UCEC); and (p) oral squamous cell carcinoma (OSCC). Figure S2: Cancer‐type specific associations between ERMP1 and the tumor microenvironment. Correlation analysis of ERMP1 expression levels with tumor microenvironment components (including tumor purity, B cells, CD8+ T cells, CD4+ T cells, and macrophages) across multiple cancer types: breast cancer (BRCA), head and neck squamous cell carcinoma (HNSC), kidney renal clear cell carcinoma (KIRC), hepatocellular carcinoma (LIHC), pancreatic adenocarcinoma (PAAD), prostate adenocarcinoma (PRAD), and thyroid carcinoma (THCA). [file HUMU-2026-7717815-s001.docx]
